# Three-dimensional analyses of vascular network morphology in a murine lymph node by X-ray phase-contrast tomography with a 2D Talbot array

**DOI:** 10.3389/fimmu.2022.947961

**Published:** 2022-11-29

**Authors:** Florian L. Schwarzenberg, Paul Schütz, Jörg U. Hammel, Mirko Riedel, Jasmin Bartl, Sharareh Bordbari, Svea-Celina Frank, Bernd Walkenfort, Madleen Busse, Julia Herzen, Christian Lohr, Clemens Wülfing, Stephan Henne

**Affiliations:** ^1^ INI-Research, Group for Interdisciplinary Neurobiology and Immunology, University of Hamburg, Hamburg, Germany; ^2^ Institute of Materials Physics, Helmholtz-Zentrum Hereon, Geesthacht, Germany; ^3^ Department of Physics, School of Natural Sciences, Technical University of Munich, Garching, Germany; ^4^ Munich Institute of Biomedical Engineering, Technical University of Munich, Garching, Germany; ^5^ Imaging Center Essen (IMCES), Electron Microscopy Unit (EMU), Medical Faculty, University of Duisburg-Essen, Essen, Germany; ^6^ Division of Neurophysiology, University of Hamburg, Hamburg, Germany

**Keywords:** B-cell follicle, lymph node vascularization, x-ray phase-contrast tomography, capillary density, lymphocyte homing, 3D model, high endothelial venules (HEVs), murine lymph nodes

## Abstract

With growing molecular evidence for correlations between spatial arrangement of blood vasculature and fundamental immunological functions, carried out in distinct compartments of the subdivided lymph node, there is an urgent need for three-dimensional models that can link these aspects. We reconstructed such models at a 1.84 µm resolution by the means of X-ray phase-contrast imaging with a 2D Talbot array in a short time without any staining. In addition reconstructions are verified in immunohistochemistry staining as well as in ultrastructural analyses. While conventional illustrations of mammalian lymph nodes depict the hilus as a definite point of blood and lymphatic vessel entry and exit, our method revealed that multiple branches enter and emerge from an area that extends up to one third of the organ’s surface. This could be a prerequisite for the drastic and location-dependent remodeling of vascularization, which is necessary for lymph node expansion during inflammation. Contrary to corrosion cast studies we identified B-cell follicles exhibiting a two times denser capillary network than the deep cortical units of the T-cell zone. In addition to our observation of high endothelial venules spatially surrounding the follicles, this suggests a direct connection between morphology and B-cell homing. Our findings will deepen the understanding of functional lymph node composition and lymphocyte migration on a fundamental basis.

## Introduction

Detailed knowledge of lymph node (LN) vascularization is crucial for understanding the complex functional properties that lead to effective immune responses against foreign pathogens. Lymph nodes are highly vascularized to maintain constant homeostasis and circulation of lymphocytes within the organ. They are positioned at strategic locations throughout the entire body for screening foreign antigens. Further on, LNs provide the optimal environment for each cell type hosted within the LN through complex subdivision into several distinct compartments to carry out all central immunological functions (1–3). Lymph nodes are spherical to bean-shaped organs, encapsulated by several layers of collagen fibrils forming a rigid barrier to the body environment (4). The LN itself can be subdivided into several different functional and morphological distinct units called lobes and the cellular composition is the same in each of these lobes. The outermost layer, the cortex, which mainly consists of B-cell follicles and interfollicular regions, surrounds the medullary regions as well as the deep cortical units (DCU) of the paracortex, where T-cells are hosted. At the interfollicular T-cell-B-cell border, the crucial interaction between B-cells and T-helper-cells occur, which ultimately activates B-cells for proliferation, somatic hypermutation and immunoglobulin production (5). The subcapsular sinus (SCS) which is located just below the capsule, constantly receives afferent lymph *via* the afferent lymphatic vessels. For each lobe one afferent lymphatic vessel drains lymph from different regions, which may result in formation of a specific antigen and cytokine microenvironment within the lobe (1, 3). As soon as the lumen of the sinus is filled with incoming lymph, subcapsular sinus macrophages start screening for foreign antigens (6, 7). On one side the SCS is bounded by the capsule and on the counter side a special type of lymphatic endothelial cells, the sinus floor lining cells, provides a barrier to the LN environment. On the opposite side, where the hilus is located, the SCS makes a transition into several medullary sinuses and lymph fluid exits the LN *via* the efferent lymphatic vessel together with the LN supplying blood vessels (8, 9). The hilus is commonly described as a narrow entry for the efferent lymphatic vessel which penetrates the capsule at the point of indentation together with one artery and one vein. Only few studies suggest that the blood supply is not restricted to one entering arteriole, but either conclusive three-dimensional (3D) data or the relation of blood vessels to the surrounding structures like the LN capsule are missing or experiments refer to species like dog and sheep (10–12). Textbook knowledge describes that one artery enters the LN *via* the hilus and immediately branches into smaller arterioles. Those arch deeper through the LN’s cortex and form a capillary bed within the whole organ. Capillaries can be classified into a continuous, fenestrated or sinusoidal type, each with a special morphology and function. Continuous capillaries have no perforations in the basal membrane which restrict the exchange with the environment to fluids, gas or very small molecules. If the basal membrane has bigger fenestrations, slightly bigger molecules can pass through the endothelial wall. Sinusoidal capillaries have the biggest pores of all types, which allow erythrocytes and smaller cells to transmigrate. The latter and most permeable type of capillaries is known to be present in lymphatic organs (13). Depending on the location, capillaries either make a direct transition into venules which merge before leaving the organ, or capillaries culminate into high endothelial venules (HEV) which form the main migration route for lymphocytes before they merge with the other venules and exit the LN (14).

HEVs are specialized postcapillary venules with a unique morphology. The vessel building endothelial cells are of high cuboidal shape which makes it easy to distinguish them from the squamous endothelium of regular venules (15, 16). HEVs are enriched in the T-cell zones of the paracortex as well as in the interfollicular spaces of the cortex next to the B-cell follicles (17). However, there is variation where HEVs transform into regular venules as they reach the medulla of the LN. Endothelial cells facing the medullary sinuses already exhibit a flattened cell shape similar to conventional venules, whereas the endothelial cells towards the T-cell zone have the typical high cuboidal shape (18). This high degree of plasticity can be explained by the multiple functions they execute. For instance, HEVs are crucial for lymphocyte homing as they provide lymphocyte transit through the endothelial wall (17, 19, 20). Further, the high cuboidal shape of the endothelial cells is most likely an adaptation to provide minimal fluid leakage into the LN during transvascular cell migration (21). This transvascular traffic of lymphocytes depends on several factors like special adhesion molecules to slow down lymphocytes at specific sites of the HEV prior their migration into the LN (22). This mechanism is crucial for the migration of naive B- and T-cells, as well as for memory T-cells and precursor conventional dendritic cells (pre-cDCs) into niches of the vessel wall to exit the bloodstream and subsequently migrate into the LN parenchyma. There is also evidence that soluble chemokines as well as antigens are transported through the conduit system to the HEVs, and thus have a direct impact on lymphocyte traffic and recruitment at HEVs (23). This conduit system is a unique structure within LNs that restricts the classic extracellular matrix to its lumen (24). This meshwork is formed by fibroblastic reticular cells (FRCs), which surround a core of several collagen fibers that are connected to numerous basement membrane molecules. The surrounding FRCs are closely interconnected to seal the conduit from the LN cortex. Even though this kind of “shielding” is crucial to prevent free antigen distribution into the LN, approximately 10% of the conduit surface is not covered by FRCs. These open spots are occupied by dendritic cells (DCs), which can reach into the conduit with their cellular extensions for uptake of foreign antigens and in return present them to naive T- and B-cells (25). Overall, the conduit system with its morphological complexity provides the structural integrity of the LN. The conduit connects the SCS with the cortical regions of the LN. Particularly, a close connection to HEVs is formed by the conduits throughout the LN (25, 26). Soluble molecules smaller than 70 kDa, which are filtered through the size exclusion principle, and can be delivered directly through the conduits which spread throughout the whole LN in a 3D manner. By transporting incoming cytokines and chemokines from the infected peripheral tissue to the HEVs, the conduit is able to mirror the inflammatory situation and enables a specific recruitment of lymphocytes from the bloodstream into the LN parenchyma *via* the HEVs (27, 28). Further, DCs that cover open sites of the conduit have access to transported antigens and can present those to naive B and T lymphocytes. This antigen presentation of residential DCs occurs much earlier than the antigen presentation from migratory DCs. Due to this early antigen presentation, the LN is set in an alert state while the second wave of antigen presentation by migratory DCs results in a proper adaptive immune response (29–32). In addition, there is growing evidence that B-cells might get in contact with their specific antigen as they remain in the perivenular zone around HEVs, which is tightly connected with the conduit, for more than an hour (33, 34).

Former studies on the vascularization of LNs often suffer from lower resolution, lack 3D data or rely on the staining of vascular vessels. Either antibodies, fluorescent dyes or casting materials like special resins are incorporated *via* perfusion of the blood vessels (10, 35–37). However, perfusion is often tricky and results in incomplete flush of the vascular system as blood clotting in the finest capillaries cannot be excluded resulting in castings of variable quality (14, 38, 39). To prevent the loss of fine structures due to preparative or methodological issues we aimed for a non-invasive method to investigate fixed LNs and its surrounding tissues in a close to native state. X-ray phase-contrast tomography with a 2D Talbot array allows for such unstained, whole mount LN and surrounding tissue imaging within two hours with only few to none special tissue preparations. Due to the short scanning time we were able to perform whole imaging sessions for four LNs in total. The synchrotron radiation based micro-computed tomography (SRµCT) scans resulted in high-resolution 3D models of the LN with a spatial resolution of 1.84 µm per pixel. This resolution is equal or even higher compared to other approaches (39–43) while also retaining a proportionally large field of view, which normally decreases drastically at high resolutions (44). Within this study, emphasis was put on the overall vascularization as well as on the capillary beds within the different compartments of the LN and the distribution of associated HEVs. Besides general morphological features, blood vessels were clearly distinguishable down to the finest capillaries. Further, we were able to reveal different capillary densities throughout the different LN compartments not only in a 3D model resulting from reconstructions of the SRµCT image stacks, but in addition statistical analyses that support the visual impressions.

## Material and methods

### Animals

Animals used for this study were 4 to 6 month old females of C57BL/6J mice. Housing and dissection of organs was carried out at the neurophysiology section of the biological department, University of Hamburg and in accordance with European Union’s and local welfare guidelines (Behörde für Gesundheit und Verbraucherschutz, Hamburg, Germany; GZ G21305/591-00.33).

### Immunohistochemistry

Inguinal lymph nodes were snap frozen with liquid nitrogen after dissection and cut into 20 µm sections on a Cryostat (Leica CM 1950) and placed on adhesive microscope slides (Histobond^®^+, Marienfeld, Lauda Königshofen, Germany). Subsequently, sections dried for 30 min at room temperature prior fixation with Acetone-Methanol (1:1). Fixation was carried out at -20°C for 5 min. Afterwards the tissue sections were blocked with 5% normal goat serum (Abcam, Cat.Nr.: ab7481, Lot.Nr.: GR3336429-15, Cambridge, UK) in 1X phosphate buffered saline (PBS, Gibco™, Cat.Nr. 12549079, Thermo Fisher Scientific, Waltham, Massachusetts, USA) with 0.1% TritonX-100 (Carl Roth, Cat.Nr.: 9002-93-1, Karlsruhe, Germany) (0.1% PBT). Slides were then washed for 2x 5 min in 0.1% PBT followed by a second blocking step with F(ab) antibody anti-mouse (Abcam, Cat.Nr.: ab6668, Lot.Nr.:GR3382555-2, Cambridge, UK) in a 1:50 dilution with 0.1% PBT at 4°C overnight. Prior to incubation with the primary antibodies (**Table 1**), another three washing steps were carried out in 0.1% PBT for 5 min each. Primary antibodies were incubated either for 2 hours at room temperature or overnight at 4°C. Subsequently, slides were washed 3x 5 min in 0.1% PBT followed by quenching autofluorescence with Vector^®^ TrueVIEW^®^ Autofluorescence Quenching Kit (Vector Laboratories, Cat.Nr.: SP-8400-15, Burlingame, California, USA) for 4 min. Afterwards slides were washed for 2x 10 min in 0.1% PBT and counterstained with DAPI as a nuclear counterstain (Cell Signaling, Cat.Nr.: 4083S, Cambridge, UK) 1:5000 in 0.1% PBT for 30 min. In slides where unconjugated antibodies were used, secondary antibodies (**Table 1**) were incubated together with DAPI. The slides were then washed 2x 10 min in 1x PBS and mounted with Neo-Mount (Merck, Cat.Nr.: 1090160100, Darmstadt, Germany). Images were recorded with ZEISS Axio Imager M2m (Carl Zeiss AG, Oberkochen, Germany) and finalized with the ZEISS ZEN 3.4 software.

**Table 1:** Details of primary and secondary antibodies used for immunohistochemistry stainings in this study.

Primary Antibodies	Host	Dilution	Company (Cat.Nr.)	Clone	Conjugation
Anti-Neurofilament, panaxonal (cocktail, purified)	Mouse	1:1000	Biolegend (837904)	SMI 312	–
Anti-CD19	Rat	1:100	Biolegend (115552)	6D5	Alexa Fluor 594
Anti-CD31	Rat	1:100	Biolegend (102516)	MEC13.3	Alexa Fluor 647
Anti-PNAd	Rat	1:200	Thermo Fisher (53-6036-80)	MECA-79	Alexa Fluor 488
**Secondary Antibodies**					
Anti-mouse IgG (H+L), F(ab´)2 fragment	Goat	1:2000	Cell Signaling (4409S)	–	Alexa Fluor 555

### Synchrotron radiation based micro-computed tomography

Inguinal lymph nodes were dissected by removing a tissue block of approximately 5 mm edge length with the LN in its center to facilitate that all blood- and lymph vessels directly at the site of the organ stay intact. After removal, the samples were fixed in 4% PFA in PBS (pH 7.4) for about 1h at room temperature and subsequently immersed in 70% EtOH. Samples were further dehydrated in a graded series of 2-Propanol and finally embedded under vacuum (-0.08 MPa) in Hard Plus 812 resin (EMS Diasum, Cat.Nr. 14115, Hatfield, Pennsylvania, USA). Fixation and dehydration of the samples resulted in tissue shrinkage of approximately 20 to 30%. The scanning setup was incorporated at the micro-tomography end-station at the imaging beamline P05 (REF) at PETRA III/DESY operated by Helmholtz-Zentrum Hereon (Geesthacht, Germany). To provide a monochromatic beam, an undulator source together with a double crystal monochromator were used. The detection system consists of a Ximea CB500MG camera with a CMOSIS CMV50000 sensor. The field of view covered by the beam within the five-fold magnification setup was about 7 mm in width and 3 mm in height. Further setup settings and scanning of the biological samples was performed according to Riedel and colleagues (45).

**Figure 1:**
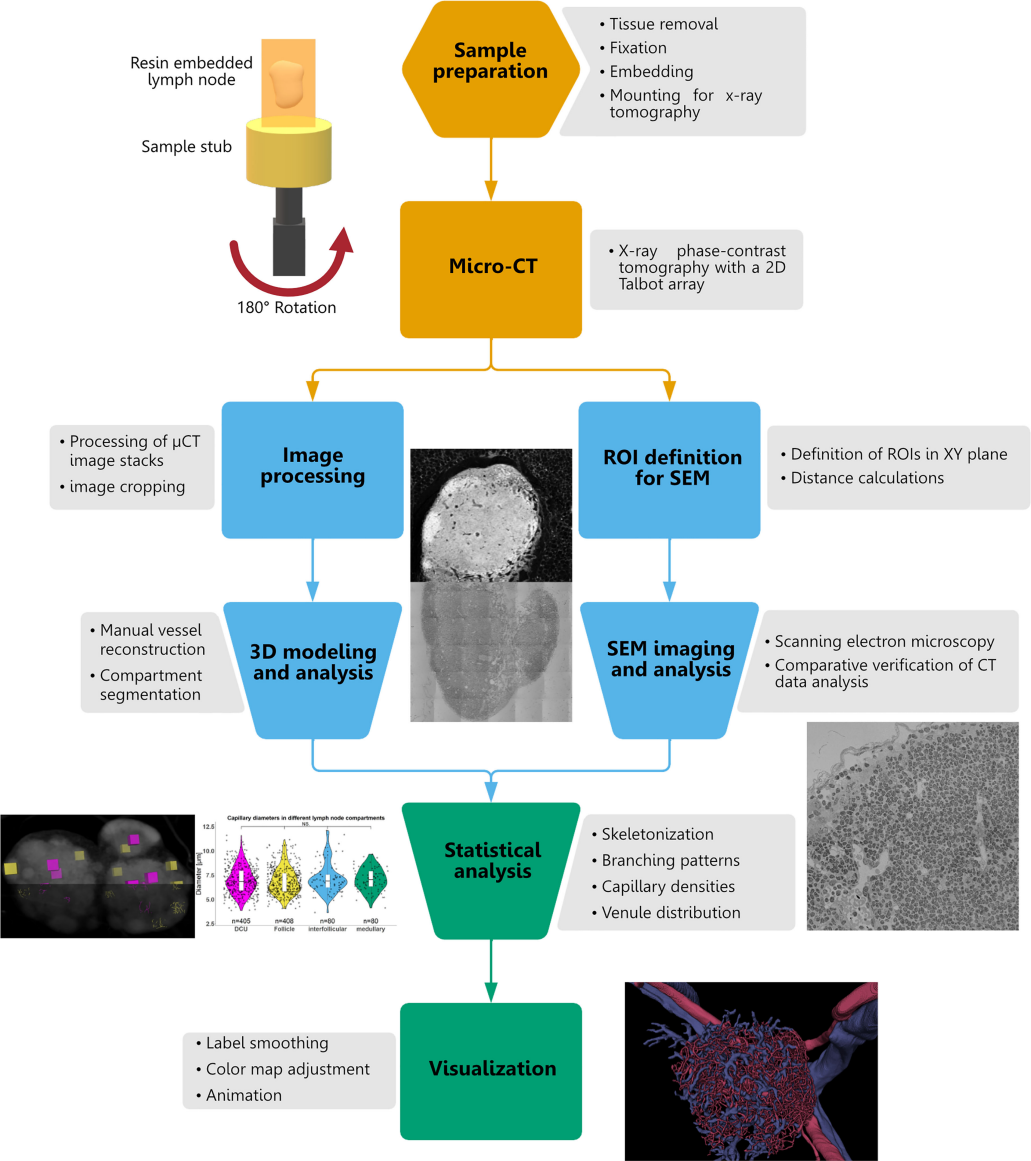
Work-flow to acquire and evaluate three-dimensional models from murine inguinal lymph nodes. The first step (orange) includes the acquisition of synchrotron radiation based micro-computed tomography (SRµCT) data by the means of X-ray phase-contrast tomography with a 2D Talbot array. For this, inguinal lymph nodes are dissected from mice and roughly but not completely freed from surrounding tissue to preserve blood and lymphatic vessel architecture. Samples are fixed with 4% PFA and subsequently dehydrated in a graded series of 2-Propanol and finally embedded into resin. Afterwards, samples are mounted on stubs and scanned in a special synchrotron setup. The second step (blue) is split up. On the one hand, SRµCT scans are further processed and cropped to reduce data size and computing power. For three-dimensional reconstruction the produced image stack is loaded into Amira/Avizo and blood vasculature as well as lymph node compartments are segmented manually. On the other hand, the embedded samples are further used for scanning electron microscopy (SEM) analyses. Here, regions of interest (ROI) are selected from the SRµCT image stack and the distance from the tip of the resin block to the ROI is calculated through the number of pixels in between and the corresponding spatial resolution. The resin block is vertically cut at the calculated ROIs and SEM is performed resulting in sections that perfectly match the xyz plane of the SRµCT for comparative analysis and verification of reconstruction. The last step (green) involves the statistical evaluation of the reconstructed networks especially by using the skeleton module of Amira/Avizo. For visualization the smoothing labels module of Amira is used and color maps are created and adjusted manually *via* the create labels colormap module.

In brief, two of the three samples were mounted in Eppendorf tubes and scanned in 70% EtOH, while the third lymph node embedded in a resin block was directly glued onto a sample holder, which was placed on an air-bearing rotational stage. The scans were acquired using a differential phase-contrast setup with a 2D Talbot array as a wavefront marker (details see Gustschin and colleagues (46). A 2D Talbot array illuminator with a period of 10 µm, a duty cycle of DC = 1/3 and a phase shift of *φ* =2π/3 was used. The scan protocols were optimized for the samples in liquid and the one in hard resin separately. For the liquid samples a photon energy of 33 keV was used, in order to reduce the dose in the liquid medium. A total of 3001 projections were recorded at a distance from sample to detector of 300 mm and an exposure time of 180 ms. For the hard resin embedded sample, a beam energy of 20 keV was used to record 1501 projections at a distance of 200 mm and an exposure time of 110 ms. All scans were recorded in continuous rotation mode. The phase retrieval was computed, using the “Unified Modulated Pattern Analysis” (47, 48), the 2D differential phase was integrated and reconstructed *via* filtered back projection. The effective pixel size in all scans was 0.92 µm resulting in a voxel size of 1.84 µm after a two-fold pixel binning in the final images (**Figure 1**).

### Visualization and statistical analysis

Acquired 16-bit image stacks of the SRµCT scans were processed and cropped in advance to the three-dimensional reconstructions with Amira/Avizo software. A volren module was used to visualize the LN’s appearances and structures of interest were manually reconstructed with the segmentation tool. Color maps were manually created for better visualization.

For reproducible statistics, we have analyzed a total of n=3 lymph nodes and defined several measuring points for each parameter investigated.

Measurements were taken with ImageJ Fiji (49) and subsequent statistical analyses were conducted with the segmentation tool in Amira/Avizo. Structures like blood vessels or B-cell follicles were segmented in Amira/Avizo and individual voxel counts for volumetric calculations were assessed through the material statistics tool. Furthermore, capillary densities in distinct compartments of the LN were analyzed in Amira/Avizo and statistically compared. In detail, a defined volume of interest (VOI) was selected, that would fit within the two compartments being the deep cortical unit of the paracortex and the B-cell follicles of the cortex with the latter compartment being the size restricting factor for the VOIs. For this task, the size of a square brush was set to 181 at a magnification of 3:1 and a spatial resolution of 1.84 µm per pixel. Subsequently, the volume was segmented for 45 pixels resulting in a defined rectangle with edge lengths of approximately 111 µm x 111 µm x 82 µm and a volume of 0.001 mm^3^. Capillaries within this volume were completely segmented and assigned to a separate material for individual volumetric calculations. For enhanced shape analysis of the distinct capillary networks the auto skeleton tool of Amira/Avizo was used. In brief, segmented labels of the capillary networks were exported using the arithmetic tool and the auto skeleton module was applied with a smoothing coefficient of 0.75. This procedure extracted the centerline of the capillaries and allowed further analysis of geometrical properties, for instance, the total and mean segment length as well as the count of branching nodes. A segment refers to the vessel between two branching events.

The diameter of the capillaries was measured for each compartment of the lymph node at several locations (at least n=80) and tested for significant alterations (Kruskal-Wallis H test). To check for differences within the capillary network (i.e., capillary density, capillary diameter, branching nodes, segment count and length per segment) among different lymph node compartments, a total of 20 boxes (VOIs) were randomly chosen for each compartment. Afterwards, each parameter was checked for significant alterations (Mann-Whitney U test) within these defined VOIs.

Branching patterns of venous vasculature were analyzed by manually assigning venules to new materials with ascending orders at each branching point downwards from the main venous trunk. In detail, at branching points where both resulting venules had approximately half the former diameter, both were added to a higher order. Otherwise, if smaller venules formed side branches without visibly decreasing the diameter, the side branch was considered a higher order while the main venule remained in the same order (10).

Graphical illustrations as well as statistical evaluation were done using RStudio v1.3.1073 (50). The graphs were constructed using the Tidyverse package v1.3.1 (51);, in particular ggplot2. Furthermore, the packages EnvStats v2.4.0 (52), cowplot v1.1.1 (53) and ggsignif v0.6.3 (54) helped to increase the data output and with the arrangement of the graphs.

### Scanning electron microscopy

Excessive resin on the sides of the sample block containing no tissue has been removed with a razor blade. The tip of the block was used as a reference point to determine the distance to the first region of interest in correlation with the SRµCT data.

The trimmed sample block was mounted on a microtome (Leica Microsystems, Wetzlar, Germany; UC7), equipped with a Diamond knife (Diatome Ltd., Nidal, Switzerland, Cat.Nr.: DH4560), for cutting semi-thin sections of 500 nm thickness. Consecutive sections floating on the water bath were collected on a piece of silicon wafer (Ted Pella Inc., Redding, California, USA; Cat.Nr.: 16006) which has been pre-treated by glow discharge (Ted Pella Inc. easyGlow) to render the surface hydrophilic. The dried sections on the wafer were post-stained for 15min in a 2% aqueous uranyl-acetate solution at room temperature in the absence of light followed by extensive washing with deionized water and repeated drying. To improve the conductivity of the section surface, a 4 nm carbon layer was deposited by carbon thread evaporation in high vacuum (Leica Microsystems, Wetzlar, Germany; ACE600).

Image acquisition was carried out on a Field Emission Scanning Electron Microscope (FESEM, Carl Zeiss AG, Oberkochen, Germany; Crossbeam 450) with an energy selective backscattered electron detector. Software automatization (v 5.2.2.85; Atlas Carl Zeiss AG, Oberkochen, Germany) for overview and detail images was used with the following parameters: accelerating voltage, 1.5 kV; probe current, 4 nA; working distance, 5.1 mm; ESB grid voltage, 750 V; pixel spacing, 200 nm (overview), 50 nm (detail); dwell time 10 µs.

### Spatial orientation within the tissue sample

**Figure 2:**
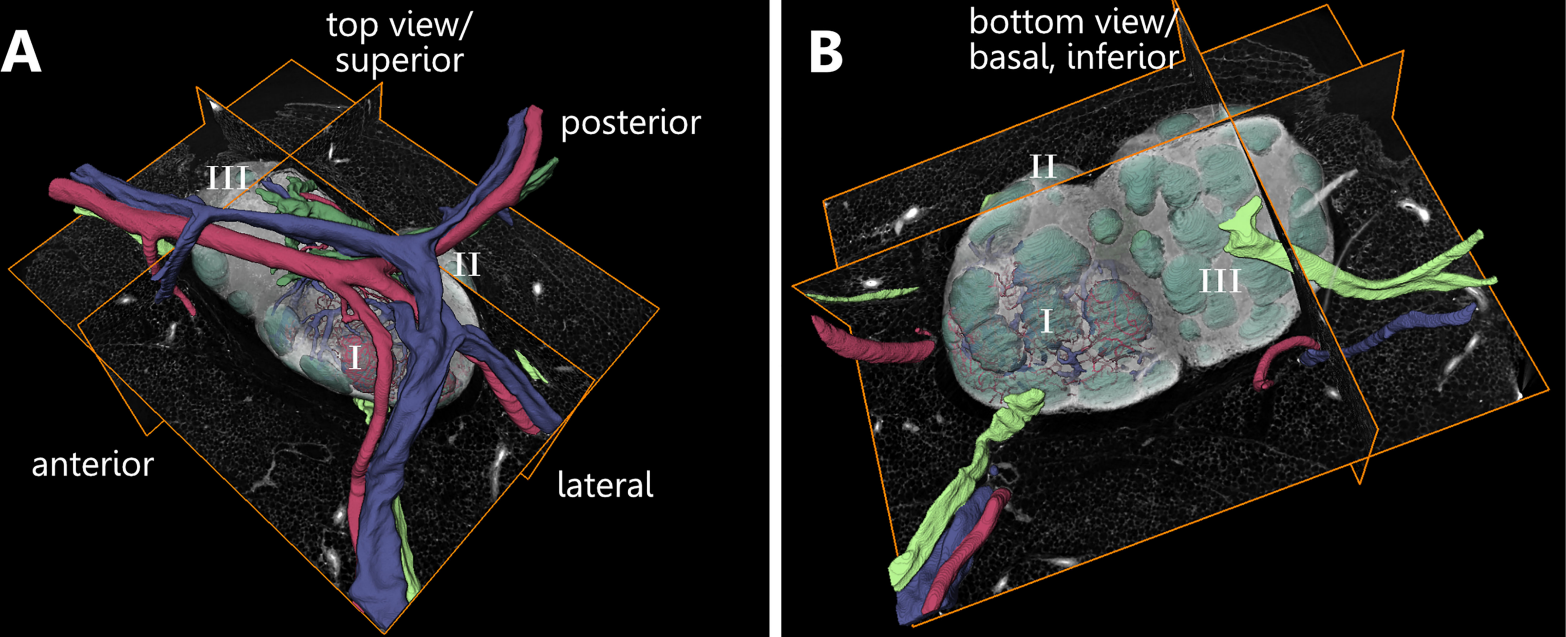
Orientational description related to the three-dimensional appearance of a murine inguinal lymph node. An animated visualization of this figure is available in the supplementary files (**Supplement Video S1**). **(A, B)** Volume rendering of an image stack acquired by means of X-ray phase-contrast tomography with a 2D Talbot array. The lymph node is subdivided into three lobes (I-III). **(A)** For enhanced comprehensibility of the figures and the model itself basic directional descriptions are established. The top view is directed towards the entrance area of blood vessels. **(B)** The bottom view of the model is directed towards the opposite side which is characterized by the presence of follicles and afferent lymphatic vessels. Afferent lymphatic vessel=light green, artery=red, efferent lymphatic vessel=dark green, Follicle=mint green, vein=blue.

For better understanding of the spatial arrangement and orientation of the LN model, we created an exemplary directional model to elucidate the constellation between different structures within LN compartments in a 3D manner. We established general spatial terminologies to properly describe the point of view onto the corresponding 3D model (**Figure 2** and **Supplement Video S1**). Relating to the hilus region, we determined the viewing angle as a top view or superior view. Further, the opposite side to the hilus will be referred to as the bottom view or basal/inferior view. Associated with the viewing angle on the model, we refer to the front view as the anterior side, the back part of the model as the posterior side, and the side part as the lateral.

## Results

X-ray phase-contrast tomography with a 2D Talbot array allows visualizing whole organ samples within a field of view of about 7 mm in width and 3 mm in height. Additionally, no complex pre-imaging tissue preparation is needed which reduces artifacts and tissue deformation or alteration to a minimum. Micro computed tomography scans generated image stacks with isotropic resolution of 1.84 µm. The surface of the LN capsule showed two visually divisible parts. The smooth appearing capsule generated higher contrast during imaging compared to a looser framework at the putative hilus region which resulted in lower contrast. Also, different compartments and structures within the LN showed clear distinguishable contrasts visible in different grayscale shades. The difference in contrast allows identifying and reconstructing structures and areas of interest like the blood vasculature in its entirety as well as afferent and efferent lymphatic vessels based on general morphological features of these structures. Within the LN, B-cell follicles could be reconstructed due to an enhanced contrast based on the high cell density inside these follicles. For verification additional scanning electron microscopy (SEM) imaging as well as immunohistochemical (IHC) stainings were performed to prove correct reconstruction and labeling of structures.

**Figure 3:**
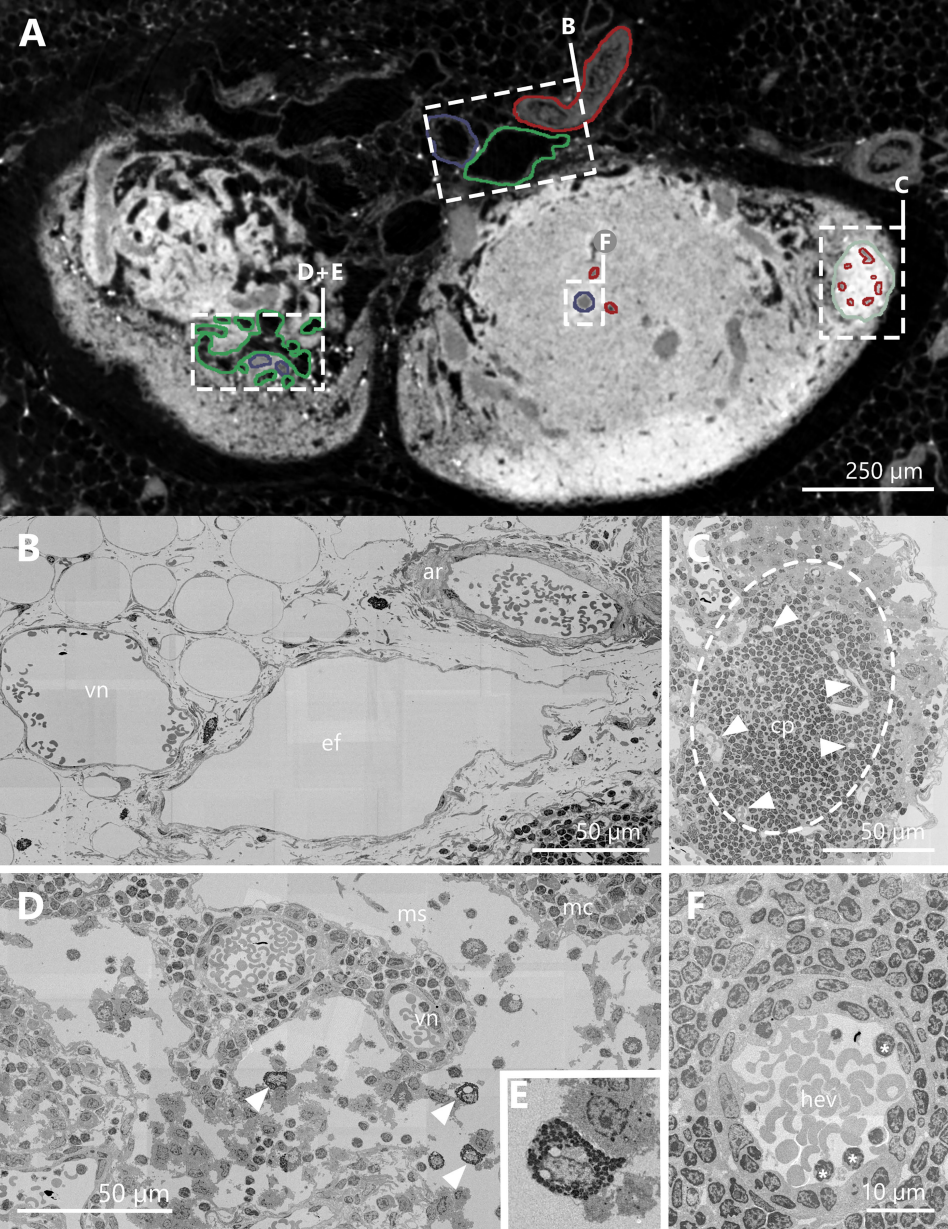
Correlation of synchrotron radiation based micro-computed tomography (SRµCT) images with high resolution scanning electron micrographs. **(A)** Single plane of an image acquired by means of X-ray phase-contrast imaging with a 2D Talbot array. The displayed section is orientated in a parallel fashion towards the hilus and projects through two lobes of an inguinal lymph node at the indentation of the hilus. For structural verification of accurate segmentation of vessels and structures, relevant objects are highlighted and compared with matching scanning electron microscopy (SEM) images. Arterial vessels and capillaries are highlighted in red, veins and high endothelial venules in blue, lymphatic vessels and medullary sinuses in green and B-cell follicles in mint green. **(B–F)** SEM images of structures highlighted in panel **(A, B)** SEM image of an artery, a vein, and an efferent lymphatic vessel in close proximity to the lymph node. The artery consists of a thick muscle layer compared to the vein. Both vessels have erythrocytes within their lumen. The efferent vessel is in structure comparable to the vein, but lacks the erythrocytes within the lumen. **(C)** SEM image of a B-cell follicle (dashed line) with several capillaries inside (arrowheads). **(D)** SEM image of the medullary region of the LN. The equivalent SRµCT images show bright white dots, which turned out to be granulocytes, most likely mast cells (arrowheads + panel E). **(E)** SEM image close up of a granulocyte. **(F)** SEM image of a high endothelial venule. The lumen is covered by the typical cuboidal endothelium. Some lymphocytes are in close contact to the high endothelial cells (asterisks). arterial system=ar, capillary=cp, efferent lymphatic vessel=ef, B-cell follicle=fo, high endothelial venule=hev, medullary cord=mc, medullary sinus=ms, venous system=vn.

The arterial vasculature was easy to identify with SEM images due to its thick smooth muscle cell layer (**Figures 3A, B**). Contrary, the venous vessels consist of a much thinner muscle cell layer (**Figure 3B**). Additionally, both systems contained erythrocytes, which allowed for a clear differentiation against lymphatic vessels (**Figure 3B**). B-cell follicles appeared in high contrast in SRµCT scans due to being densely packed with B-cells (**Figure 3C**). Tiny bright spots in SRµCT images trace back to granulocytes, putatively mast cells strategically located around blood and lymphatic vessels (55) within the LN parenchyma (**Figures 3D, E**). The vessel lumen diameter measured for SRµCT data and SEM data was identical, which confirms our reconstruction. Furthermore, the correct reconstruction of high endothelial venules (HEV) was proven through SEM images which revealed the typical cuboidal shape of their endothelial cells (**Figure 3F**).

### Redescription of the hilus as a region rather than a definite point

**Figure 4:**
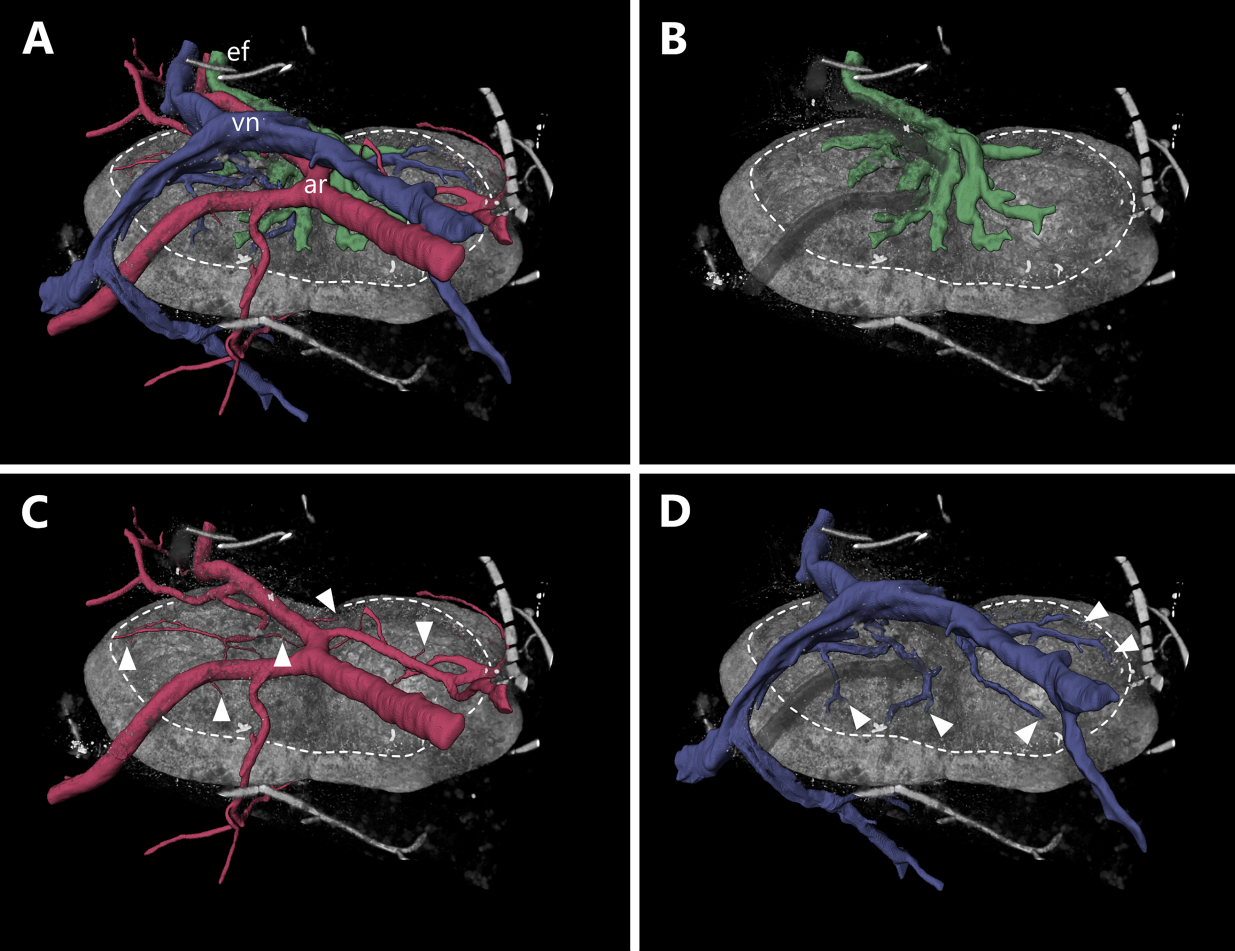
Entrance and exit area of blood as well as lymphatic vessels of a murine inguinal lymph node. An animated visualization of this figure is available in the supplementary files (**Supplement Video S2**). **(A–D)** Volume rendering of an image stack acquired by means of X-ray phase-contrast imaging with a 2D Talbot array. The lymph node is displayed in an anterosuperior view. **(A)** Several vascular structures like the organ supplying artery, the draining vein as well as the efferent lymphatic vessel are highlighted. The lymph node itself (gray volume) features an ellipsoid to slightly bean-shaped appearance and measures roughly 2150 µm x 1200 µm x 750 µm (L x W x H). The entrance and exit area of vessels (encircled by dashed line) makes up to one third of the overall organ surface and is visually distinguishable from the rest of the capsule being less dense. The main arterial and venous trunk parallel each other while the entry and exit sites for arterioles and venules do not (compare **C, D**) **(B)** Medullary sinuses merge to the efferent lymphatic vessel in the indent of the organ (Hilus). **(C)** One big artery trunk course above the hilus region, where few small arterioles are branching off and entering the lymph node at multiple points (arrowheads). **(D)** Many venules emerge from several sites (arrowheads) of the upper surface to converge to a big venous trunk. arterial system=ar (red), efferent lymphatic=ef (dark green), venous system=vn (blue).

Consistent with a former report from von Andrian (10), we identified multiple entry points for the arterial system, as well as several venous arches leaving the LN over a large area. These findings are in contrast to regular textbook schematics of LNs. Classically, the hilus is described as a single entry/exciting point combined for the arterial supply, the venous drainage and the emerging efferent lymphatic vessel. Here, we clearly show a putative hilus region which makes up approximately one third of the overall LN surface. This area can be clearly distinguished from the dense fibrous capsular structure around the LN with a less dense connective tissue framework resulting in less contrast during imaging (**Figure 4** and **Supplement Video S2**).

Interestingly, the arterial and venous system only parallel each other until they are in close proximity to the LN, from where they take separated routes towards the LN with unique entering/exiting patterns for both systems (**Figure 4A**). Arterial supply is supported by arterioles rather than bigger arteries. Close to the LN several arterial branches deviate from the main supplying artery and split into multiple arterioles which enter the LN at several sites (**Figure 4C**). Multiple venules emerge from deeper parenchymal regions of the LN through the medulla out of the LN and merge together with the main draining venous trunk (**Figure 4D**). Small lymphatic vessels emerge from medullary cords, comparable to the exit pattern of the venous system, and fuse together to a large efferent lymphatic vessel which leaves the LN. The small emerging lymphatic vessels are intermingled with the entry/exiting point from the arterial and venous system (**Figures 4B, D**).

### Elucidating the lymph node gross anatomy

**Figure 5:**
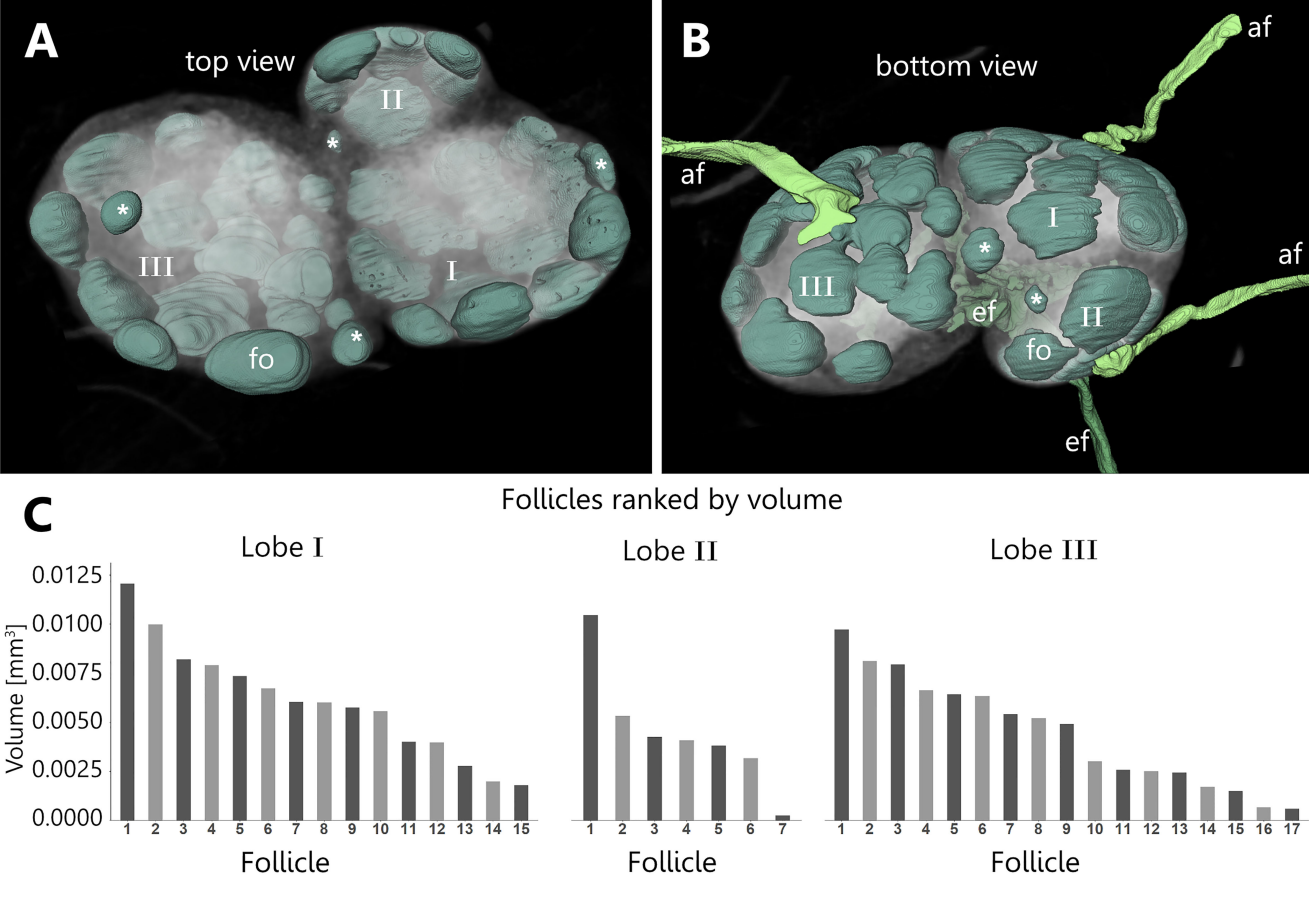
Spatial distribution and volumetric measurements of B-cell follicles within the cortex of an inguinal lymph node. An animated visualization of this figure is available in the supplementary files (**Supplement Video S3**). **(A, B)** Volume rendering of an image stack acquired by means of X-ray phase-contrast imaging with a 2D Talbot array. The lymph node can be visually divided in three distinct lobes (I, II, III) of different sizes. The B-cell follicles (mint green) are spherical to ellipsoid in shape. The follicles of each of the three lobes account for 3 to 9% of the total lymph node volume. Follicles facing towards medullary regions decrease in size (asterisks). **(A)** Top view on the three lobes of the lymph node in low opacity. The follicles are distributed to the basal area and are absent in the hilus region. **(B)** On the basal side each lobe is supplied by a separate afferent lymphatic vessel (light green) while one big efferent lymphatic vessel (dark green) drains the lymph node at the hilus region. **(C)** Follicle volumes were calculated through their respective voxel counts. Follicle volumes are rather heterogeneous and independent from the size of the lobes. Volumes are within a similar size range while each lobe features at least one comparable large follicle. afferent lymphatics=af (light green), efferent lymphatic=ef (dark green), B-cell follicle=fo (mint green).

The follicles of all three lobes of a LN were manually reconstructed to elucidate the overall morphology as well as the correlation between lobe size and follicle count. The examined LN can be separated into three distinct lobes of different sizes. Each lobe receives lymph fluid *via* its own afferent lymphatic vessel. B-cell follicles are arranged towards the basal and lateral regions where also the afferent supply comes in while the follicles are absent in the hilus region (**Figures 5A, B** and **Supplement Video S3**). The amount of follicles per LN varies between 26 and 39 and displays a dependency on the size of the corresponding lobe with the highest count for the biggest lobe and the lowest count for the smallest lobe, respectively (**Figure 5C**). Sizes of follicles range from 0.0117 mm³ to 0.0002 mm³ (**Figure 5C**), while larger follicles tend to be ellipsoid in shape and smaller follicles are generally spherical in shape (**Figures 5A, B**). Among the differently sized lobes the size range of follicles is comparable (**Figure 5C**). Interestingly, most B-cell follicles are in close contact with at least one other and some follicles seem to be fused to a certain degree (**Figure 5B**). Therefore, the interfollicular space is overall small (**Figure 5B**). B-cell follicles are always found in close contact to the LN’s capsule, while the follicle size generally decreases towards medullary margins. Overall, the total follicle volume of each individual lobe takes up about 3 to 9% of the total LN volume (**Figure 5**). In general, we found a highly variable gross anatomy for murine inguinal LNs with lobe counts ranging from one to three while in some cases lobes are separated to a degree where they form potentially individual LNs (data not shown). In addition, alterations in the neural innervation could be detected although this study did not focus on innervation pattern, as the resolution is not high enough to reveal finest axons. However, we were able to reconstruct a putative artery-associated nerve bundle with a diameter of approximately 50 µm in one of our investigated LNs. We would like to explicitly point out a special anatomical feature, namely that the nerve tunnels through the investigated LN (**Supplement Figure S1A**). Although this is not common in all inguinal LNs, this finding could be reproduced in IHC stainings (**Supplement Figure S1B**). Based on diameter measurements, the nerve seems to consistently have a diameter of around 50 µm before and after entering the LN, which suggests that the nerve does not completely branch into smaller nervous fibers, but due to the limited resolution it is at least possible that a few small diameter axons may split off. If this nerve is solely associated with arterial blood supply or if it also contains sensory fibers for explicit communication within LNs, which are currently under investigation, is unclear (56–58).

### Breakdown of the venous system

**Figure 6:**
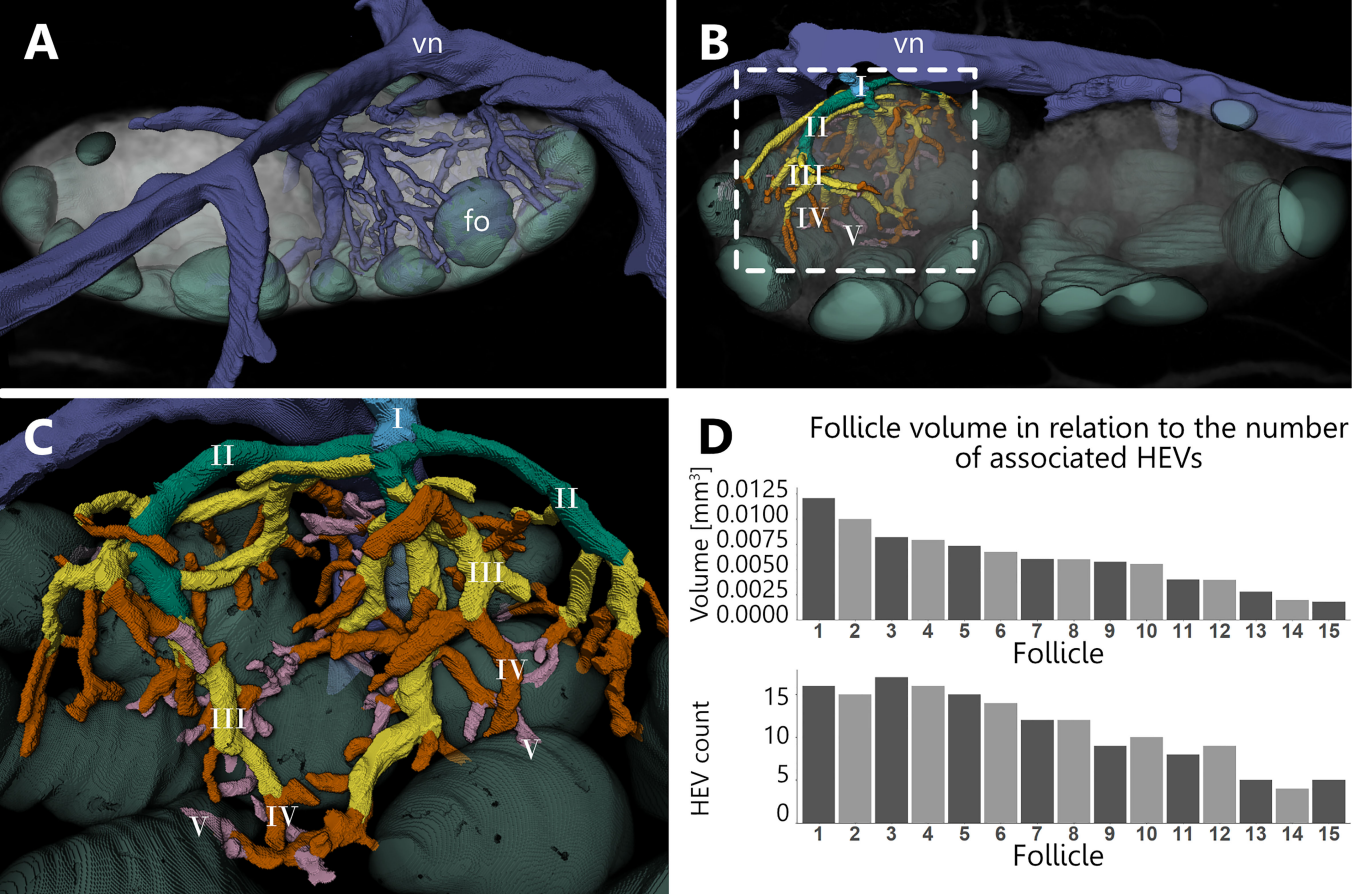
Branching pattern of the venous system and high endothelial venule (HEV) count. An animated visualization of this figure is available in the supplementary files (**Supplement Video S4**). **(A–C)** Volume rendering of an image stack acquired by means of X-ray phase-contrast imaging with a 2D Talbot array. **(A)** Anterior view. Overview on the distribution of the venous system for lobe I (see **Figure 5**). The venous system projects throughout all compartments despite the follicles. **(B)** Posterior view. Branching pattern of one of the main arches of the venous system is color coded, resembling five different orders (I-V). Venules of the order III to V are corresponding to HEVs which are often seen closely associated with B-cell follicles. **(C)** Close-up view on the branching pattern depicted in panel **(B, D)** For each follicle from lobe I, the total count of HEVs was determined which drain the corresponding capillary system of the follicles. With decreasing follicle volumes, the individual HEV counts show a declining trend. B-cell follicle=fo (mint green), venous system=vn (blue).

The venous system serves a fundamental role for LN function, since specialized venules with cuboidal shaped endothelial cells, provide the main migratory route for lymphocytes into and out of the LN. We revealed the localization of the entire venous branching pattern in relation to the complex compartmentation within murine inguinal LNs. Through manual reconstructions of the venous branches inside one lobe of a LN, we generated a model which allows for detailed spatial analysis (**Figure 6** and **Supplement Video S4**). At the T-cell/B-cell border just around the B-cell follicles, small venules occur and merge together to form the main draining venous trunk which returns blood to the main blood stream (**Figure 6A**). We categorized the branching pattern of the venous system starting with first order venules, which are directly connected to the leaving main venous trunk. From these first order venules, we followed the branching course further into the LN’s parenchyma. After each branching point, we defined a new order of venules. In total, five orders of venules were identified during analysis (**Figure 6B**).

In detail (**Figure 6C**), first order branches are primarily present outside the LN. These branch into two or more second order branches which run across the border of the LN parenchyma and enter the medulla at separate locations, partly projecting deep into the LN. Subsequently, third order branches split off towards the medullary as well as paracortical basal regions of the LN. The following fourth and fifth order branches are exclusively located in the DCU and interfollicular region and mostly protrude to the border of the B-cell follicles. Capillaries emptied primarily into venules of the orders III-V inside of the medulla as well as DCU and interfollicular regions, but occasionally also in second order venules. Approximately half of the venous volume is distributed among the last three orders. In addition, high endothelial venules are not homogeneously distributed within the lobe. Venules of the order III to V within the DCU showed a lower abundance in comparison to the surrounding venules at the medullary margins and the interfollicular regions directly at the B-cell follicles. In that regard, both, venule segment counts obtained through skeletonization and volume calculations, display a value approximately two times higher at the medullary margins and interfollicular regions than in the DCU (**Supplement Table S1**). Based on the inner and outer diameter of the venules, we identified putative high endothelial venules, which were additionally confirmed in SEM (**Figure 3F**). The typical appearance for those HEVs was only found for venules of the order III to V. Further, we investigated a potential correlation between follicle volume and an associated HEV count. The total number of HEV directly connected to the capillary beds inside the follicles was measured and set in relation to the total volume of the investigated follicle. We found that with decreasing follicle volume, the number of associated HEVs declines as well (**Figure 6D**).

### Spatial analysis of the transition from capillary to high endothelial venule

**Figure 7:**
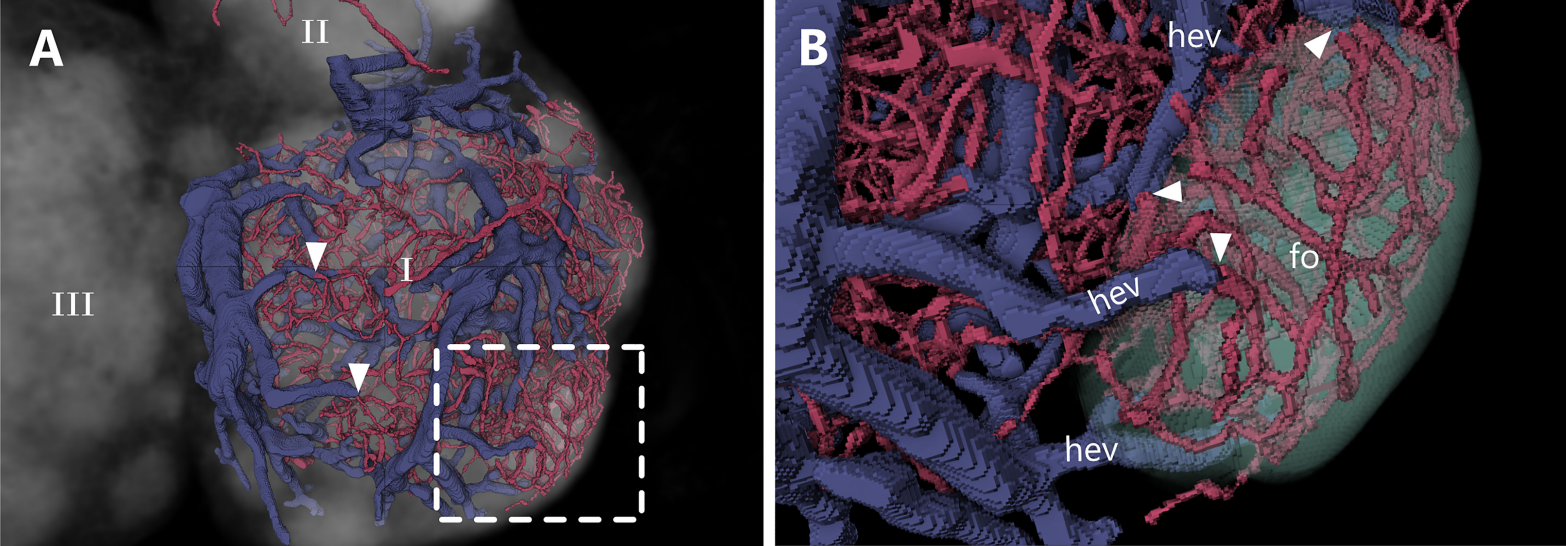
Transition from capillary beds to high endothelial venules (HEVs) at the B-cell follicle margin. An animated visualization of this figure is available in the supplementary files (**Supplement Video S5**). **(A, B)** Volume rendering of an image stack acquired by means of X-ray phase-contrast imaging with a 2D Talbot array. The lymph node is displayed in a top view. **(A)** Capillarization and venule distribution of one lobe (I). Transitions of capillaries into HEVs are clearly visible due to an abrupt change in vessel diameter with a ratio of 1:3 (arrowheads). Greater magnification in panel B (dashed line). **(B)** Close up shows a dense capillary bed within a B-cell follicle which gets drained by multiple HEVs. HEVs are closely located at the margin of the follicle. B-cell follicles=fo (mint green), high endothelial venules=hev (blue).

Despite the well described localization and morphology of HEVs, their spatial orientation in a 3D context is still missing. With our approach, we were able to identify the exact location of the point of transition from capillaries to HEVs. For a better understanding, the entire blood circulatory system of the DCU, the interfollicular regions as well as three follicles within one lobe were reconstructed exemplarily. B-cell follicles are completely devoid of venules, especially HEVs, but consist of a dense capillary network without bigger arterioles (**Figure 7** and **Supplement Video S5**). These capillaries directly transit into HEV throughout the follicular margin, with an abrupt increase in vessel diameter with a ratio of approximately 1:3 (**Figure 7B**). As mentioned in the previous section, the B-cell surrounding HEVs can be categorized as order III to V venules. Every follicle is surrounded by HEVs with a tendency for higher total HEV count associated with larger follicles.

**Figure 8:**
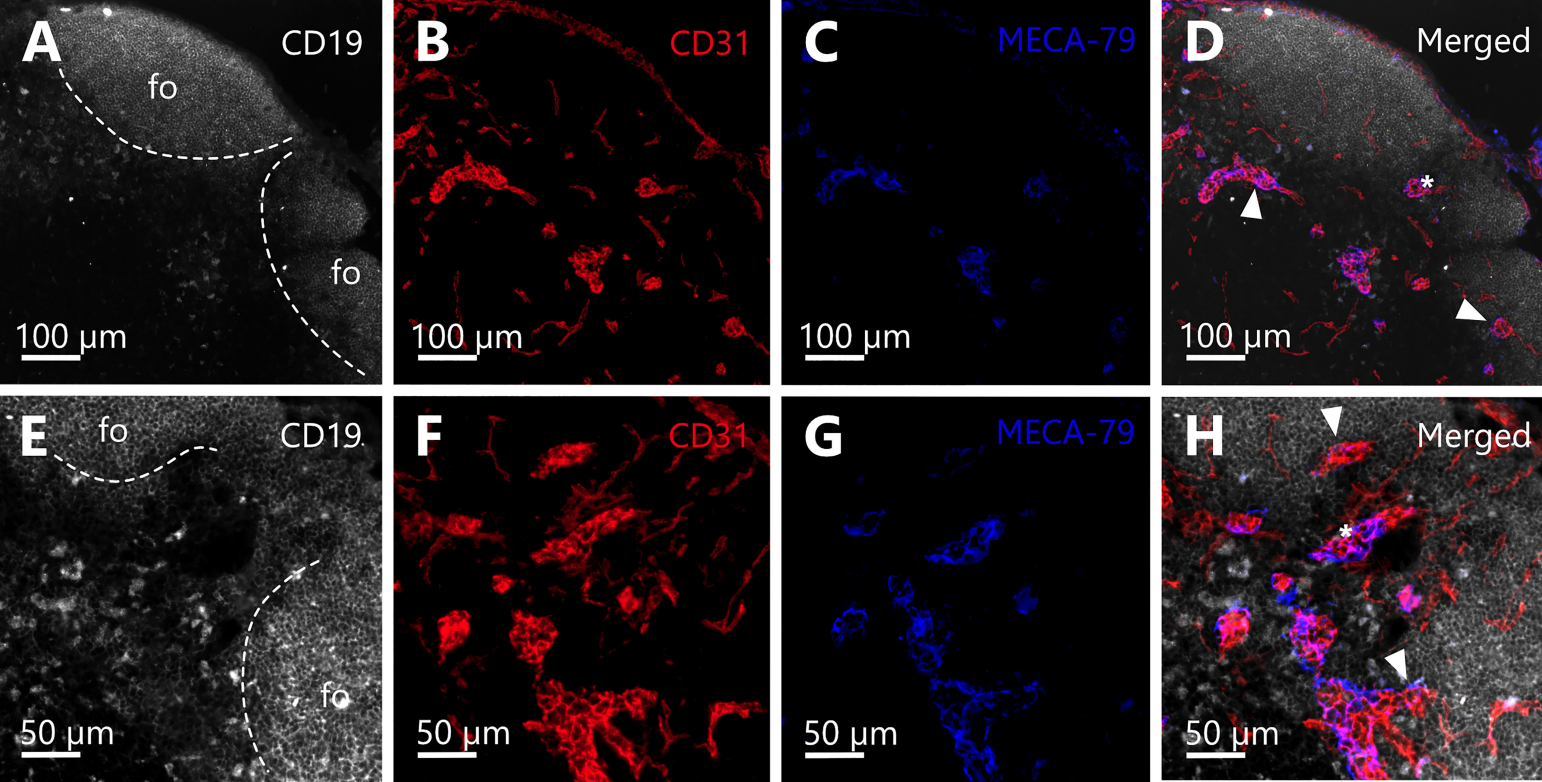
Immunohistochemical staining confirms the spatial arrangement of high endothelial venules (HEV) in close proximity to B-cell follicles. **(A–H)** Cross sections of the deeper parenchyma and cortex of a murine inguinal lymph node are depicted. B-cell follicles (fo) in the periphery of the organ bordering the lymph node capsule. **(A–D)** Immunohistochemical (IHC) staining against CD19 (white), CD31 (red), and MECA-79 (blue) with a magnification of 10x. **(E–H)** IHC staining against CD19 (white), CD31 (red), and MECA-79 (blue) of the same region of the lymph node as displayed in A-D but a different slide (approx. 20 µm deeper within the organ) and with a magnification of 20x. **(A, E)** Anti-CD19 antibody staining reveals B-cell follicles (dashed line). **(B, F)** Anti-CD31 antibody staining elucidates the blood vasculature of the lymph node and clearly shows that follicles are devoid of bigger blood vessels, yet exhibit dense capillary beds. **(C, D)** Anti-PNAd (MECA-79) staining identifies the HEVs of the venous system. **(D, H)**: Merge channels reveal the spatial relation of HEVs to B-cell follicles. Several HEVs can be found in close proximity to B-cell follicles (arrowheads). Between two follicles within the interfollicular region the course of one HEV can be recognized (asterisks in **D, H**).

Further, to prove the correct identification of spatial relations of HEVs and follicles, we performed IHC staining with structure specific antibodies against B-cells (CD19) to depict the follicles, blood endothelial cells (CD31) to reveal whole blood vasculature, and the distribution of HEVs (PNAd/MECA-79). In addition to the 3D reconstructions from the SRµCT images, the IHC staining confirmed the arrangement of HEVs close to the B-cell follicle borders and in the interfollicular regions as well as the presence of capillaries within the follicles (**Figure 8**).

### Identification of differences in the density of capillarization between lymph node compartments

**Figure 9:**
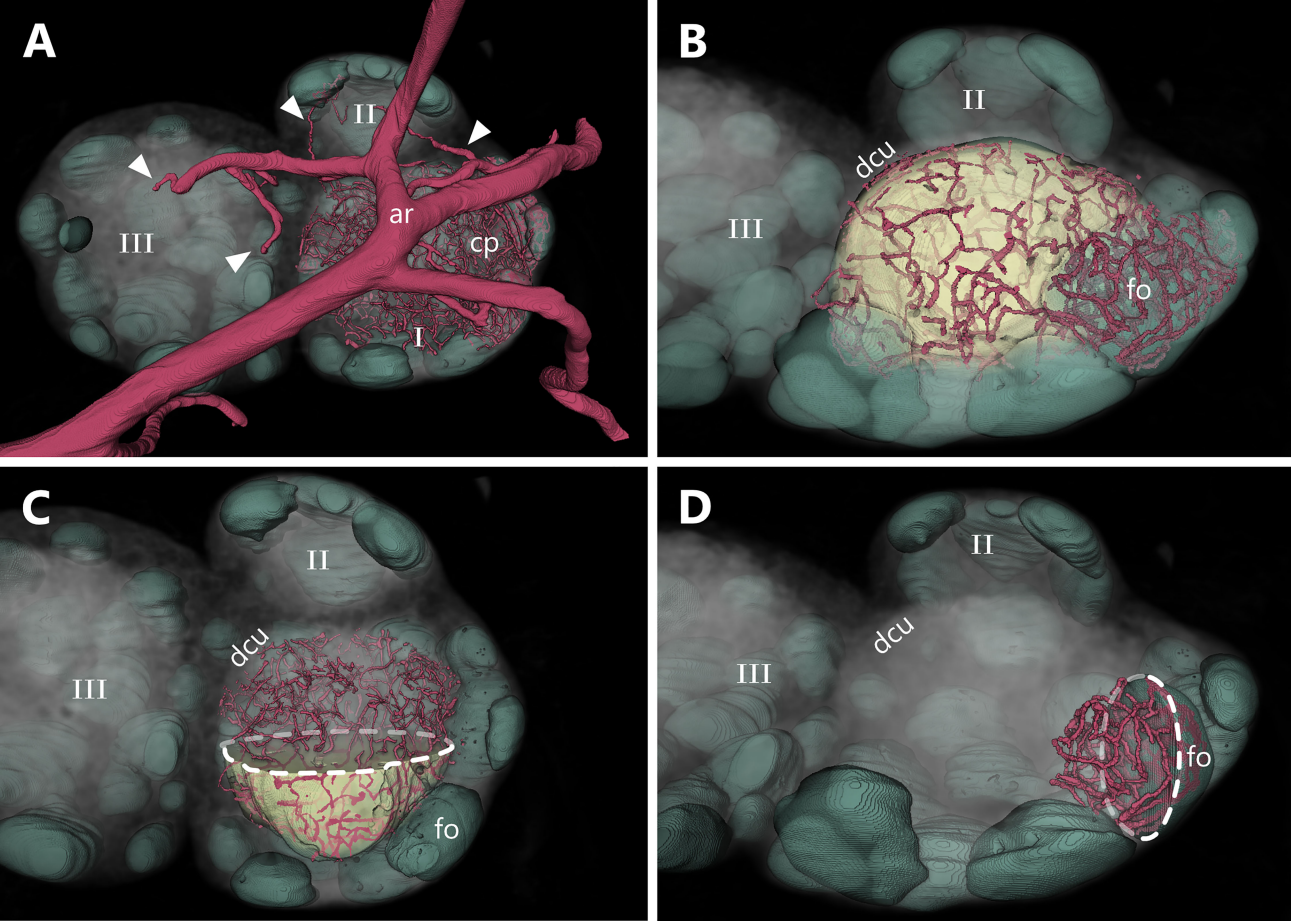
Capillary density between different lymph node (LN) compartments. An animated visualization of this figure is available in the supplementary files (**Supplement Video S6**). **(A-D)** Volume rendering of an image stack acquired by means of X-ray phase-contrast imaging with a 2D Talbot array. The LN is displayed in a top view. **(A)** Overview of the arterial supply of lobe (I) The main arterial trunk feeds multiple arterioles (arrowheads) which further branch into capillary beds throughout the LN. **(B)** Comparison of capillary density between the deep cortical unit (yellow) and the follicles (mint green) inside lobe (I) For better visualization, the main arterial trunk and the corresponding arterioles are not displayed. **(C)** To achieve better visualization of capillaries inside the deep cortical unit, the upper part of the reconstructed material is blanked out (dashed line). **(D)** To achieve better visualization of capillaries inside the B-cell follicle, the left part of the reconstructed follicle material is clipped (dashed line) as well, allowing a better view on the capillary bed inside the follicle. In comparison to the deep cortical unit (panel C), the follicular capillarization appears to be denser. Arterial system=ar (red), capillary system=cp (red), deep cortical unit=dcu (yellow), B-cell follicles=fo (mint green).

Reconstruction of the arterial supply revealed a comprehensive capillary framework within the different LN compartments. Directly after penetrating the LN’s capsule, the arterioles further branch into capillaries (**Figure 9A**). These capillaries form a dense meshwork throughout the whole organ including the DCU and the B-cell follicles (**Figure 9** and **Supplement Video S6**).

**Figure 10:**
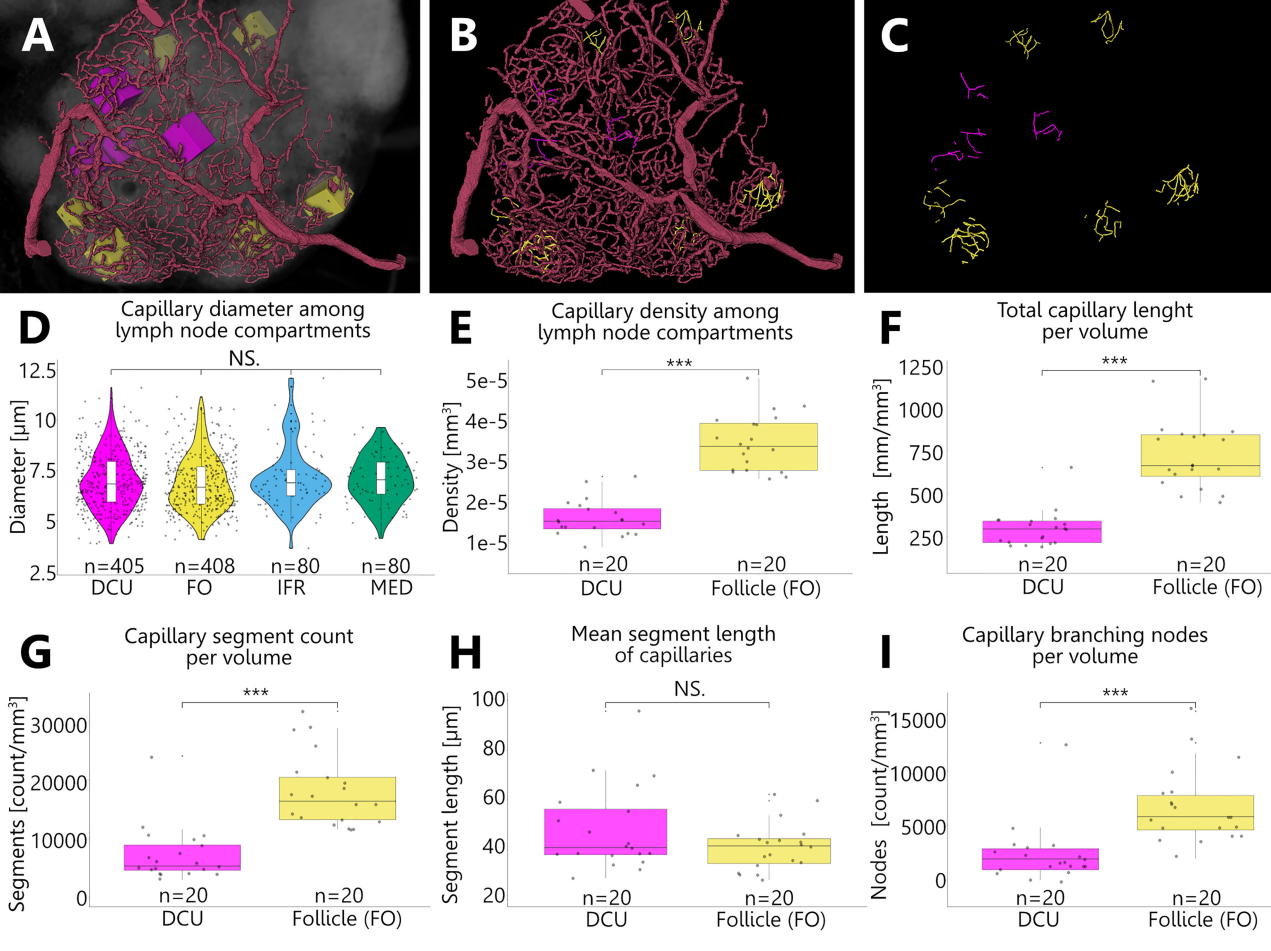
Revealing differences in capillary densities among different compartments of an inguinal lymph node. **(A–C)** Volume rendering of an image stack acquired by means of X-ray phase-contrast imaging with a 2D Talbot array. Depicted is a top view on the manually reconstructed blood supplying arterioles, which give rise to a nested capillary network in one lobe **(I)**. This network projects throughout different compartments including the deep cortical unit (DCU) as well as the follicles (FO), interfollicular regions (IFR) and the medulla (MED). For reproducible statistical quantification of capillary densities, defined volumes of interest (VOI) (boxes in panel A) were placed randomly within these compartments and the contained capillaries were completely reconstructed. **(A)** For capillary density quantification magenta boxes were placed in the DCU and yellow boxes within B-cell follicles respectively. **(B)** VOI boxes from Panel A are masked out and reconstructed capillaries are highlighted in the corresponding color. The magenta capillaries from the DCU give the visual impression that they are less dense compared to the yellow capillaries from the follicles. **(C)** Skeletonization of the reconstructed capillaries within the VOIs was executed to collect additional statistical data. **(D)** To ensure comparability of volumetric measurements between the capillary networks of different compartments capillary diameters were checked for alterations. Capillary diameters do not differ significantly among the analyzed compartments (Kruskal-Wallis H test). NS.=not significant, P>0.05. **(E–I)** The shape analysis through skeletonization allowed the examination of the geometrical and topological properties of the capillary network, like length, segment count or branching of vessels. For the DCU as well as the follicles a total of 20 VOIs (n=20) were investigated among three inguinal lymph nodes. The data was statistically analyzed using the Mann-Whitney U test as data sets were not normally distributed. ***P<0.001, NS.=not significant, P>0.05. **(E)** Volumes (mm^3^) of the capillary materials of the respective VOI boxes were calculated through their voxel counts. The capillary density within the follicles is significantly higher by the factor 2.2 compared to the DCU. **(F)** Comparison of total lengths per volume (mm/mm^3^) show a significant difference between the capillary network of the DCU and that of the follicles. On average the follicular capillaries show a 2.5 times higher value compared to the capillaries in the DCU. **(G)** Within the follicles the capillary network exhibits 2.5 times more segments than the capillary bed of the DCU. **(H)** Mean segment lengths of capillaries in the DCU are on average slightly longer than follicular capillaries but statistically comparable in length. **(I)** Branching events of capillaries within the follicles are three times more frequent compared to the capillaries of the DCU.

To analyze the capillary density of B-cell follicles as well as for the deep cortical unit, we measured the capillary volume within defined rectangular areas which were randomly positioned inside these compartments. In a similar approach to Jafarnejad and colleagues (38), we analyzed the capillary meshwork of both compartments by selecting 20 defined VOIs which were distributed among three LNs (**Figures 10A–C**). Capillary diameters measure 7 µm at average and do not differ significantly between the different compartments of the LN (**Figure 10D**). This ensures the comparability of volumetric measurements. Capillary densities were measured by means of three approaches. First, the sheer volume was calculated *via* the corresponding voxel counts (**Figure 10E**). Second, through skeletonization a compact representation of the geometrical features of the capillary network allowed the calculation of the total vascular length per volume (**Figure 10F**). Third, capillary segment counts per volume were determined (**Figure 10G**). All three approaches demonstrate a higher degree of capillarization of the B-cell follicles compared to the DCU by the factor of 2 to 2.5. While the mean segment length of capillaries within both compartments does not differ much (**Figure 10H**), branching events in B-cell follicles were three times more frequent than in the DCU (**Figure 10I**). This also verifies the significantly increased segment count within the B-cell follicles. These different approaches underline each other and support the observation of higher density of the capillary networks within follicles.

## Discussion

Revealing the LN vascularization in great detail is crucial for functional analysis of the immune system and understanding the course of infections. Tackling this task with murine LN opens the opportunity to apply findings to the human body due to the model character of the murine system. Visualization of the vasculature in an organ-wide scale is often limited by the methodical approach or by the resulting data sizes. On the one hand, a special focus on the vasculature only, excludes information about the overall spatial relation with other LN structures. On the other hand, a whole organ analysis results in a hard to process data size and often require elaborate labor and computational power. With our new methodical approach, we were able to visualize the vasculature complexity of a whole LN without losing the spatial relation to LN compartments nor the general morphology.

### Challenges for in-depth blood vasculature network analysis in lymph nodes are overcome by X-ray phase-contrast tomography with a 2D Talbot array

There are many different approaches for functional and structural elucidation of LN vasculature. One of the simplest procedures is the histological examination of tissue sections. In common approaches, vessels are, on the one hand, strongly contrasted by perfusion with dyeing solutions like alcian blue (59), india ink (60, 61) or colloidal carbon (11, 14, 62). On the other hand, vessels can be visualized using immunohistochemistry and fluorescence microscopy (10, 35, 37, 39). While providing a time-, work- and cost-efficient visualization of blood vessels, 2D image content is often not sufficient for in-depth functional examinations. A methodological advancement is achieved by vascular corrosion casting for initial 3D visualization. Based on perfusion with plastics or resin and subsequent dissolving through corrosive chemicals like potassium hydroxide, a cast of whole organ vasculature is generated that allows further analysis *via* electron microscopy and µCT scanning (14, 38, 44, 60). However, previous studies reported blunt ends of capillaries in corrosion casts, indicating that perfusion is not able to reach into the finest capillaries (14, 38). In that regard, the presence of arteriovenous shunts that short-circuit the capillary networks (63) could pose a problem for proper perfusion in general. Kelch and colleagues (39) established a procedure that is based on vessel perfusion with fluorescent lectin and following iterated confocal imaging which allows whole organ analysis of several cubic millimeters at approximately 2 µm resolution. Their method enables extensive statistical evaluation of vascularization at the cost of high computational power. Unfortunately, this method lacks the relation to the structural composition of the LN itself as only blood vasculature is visualized in 3D. This complicates the exact identification of LN subregions to establish a functional context. Other modern approaches include optical projection tomography (OPT) and selective plane illumination microscopy (SPIM), which were, for instance, able to identify spatial correlations between HEVs and B-cell Follicles as well as between HEVs and dendritic cells in LNs (35, 37), but the achieved visualizations partly lose 3D context. Although this procedure allows in-depth spatial structure elucidation, disproportionate working effort restricts the method to analysis of a single LN, and cutting as well as alignment artifacts reduce the overall quality of the resulting model. Since there is growing molecular evidence for functional connectivity and dependency of blood vasculature distribution within the highly subdivided LN (33, 64, 65), high-resolution methods are required that incorporate the aspects of whole blood vasculature acquisition with a spatial relation to the LN gross anatomy in a 3D manner as well as analysis of multiple specimens in a proportionate time frame.

### X-ray phase-contrast imaging with a 2D Talbot array allows near to native whole lymph node analysis with high throughput potential

In general, the application of X-ray imaging in biological soft tissue samples is challenging, as these consist of cells featuring mostly low-atomic-number elements. Therefore, contrast media, for example, of iodine or gold basis, are needed to compensate for the lack of tissue differentiation (66). However, this requires additional preparation steps and the selection of optimal media for the individual cause which altogether results in further deviation from the LN’s native condition. The approach of X-ray phase-contrast imaging overcomes these issues, as a phase shift is achieved through a 2D Talbot array allowing for enhanced differentiation of atomic electron densities even within unstained soft tissues (45, 46). With the given setup the LNs do not need any additional preparation steps beyond the fixation in order to produce whole organ imagery, where blood vessels down to the capillaries, medullary sinuses, B-cell follicles and even larger nerves are easily recognizable at a spatial resolution of 1.84 µm. Thus, unlike other already mentioned methods this approach is not limited to the vascular system only, but enables the establishment of a functional context between most of the present structures and compartments. Although sample scanning within ethanol poses the risk of air bubbles obstructing the scan as well as the occurrence of motion blur which slightly reduces the overall quality of the scan, this can be easily bypassed by embedding in resin. This ensures a low error rate and produces imagery with little to none artifacts at short scanning time of approximately two hours. In order to utilize the full high throughput potential, further optimizations are still needed as of right now reconstruction is quite time consuming. A semi-automatic to automatic approach is desired as it would allow statistical quantification of whole vascular reconstructions down to the finest capillaries of multiple specimens, also as a comparative approach, for instance, with mesenteric organ draining LNs or infection models. On top of that it is even possible to scan larger samples as the initial field of view of approximately 7 mm width and 3 mm height can be stacked on top of each other in multiple scans and subsequently computed back to one single imagery. In addition, it is possible to reinvestigate samples at the SEM in a high resolution (50 nm pixel size and below) and further align these sections to perfectly match the SRµCT image for verification and further analysis (**Figure 3**).

### Multiple blood supplies - redescription of the hilus in murine inguinal lymph nodes

Classically, murine inguinal LNs are described as spherical to bean-shaped organs with a single, definite entry/exit point for the arterial and venous system as well as for the efferent lymphatic vessel. This narrow opening is said to be located at the indentation of the organ. Contrasting this accepted textbook knowledge, our high resolution SRµCT scans revealed a different morphology. Over a surface area of approximately one third of the whole LN surface, multiple arterioles enter and multiple venules exit the LN, respectively. With intravital microscopy, von Andrian (10) convincingly revealed a similar morphology. He investigated the microcirculation in murine LNs and presented an arterial blood supply which spreads over the entire upper surface of the investigated inguinal LN. Despite this, similar results for dog and sheep LN support these findings (11, 12) yet, the general idea of the hilus as a definite point remained untouched over the years. With the 3D data provided within this study we suggest defining the hilus not as a definite point but rather a large area where blood- and lymphatic vessels enter and emerge, respectively. Still, a functional explanation of why the supply route of the blood vasculature for the LN spreads over a larger area than previously recognized remains to be elucidated. Given the fact that during inflammation the whole LN expands three times its initial volume (37), it is feasible to argue that despite overall volume expansion and proliferation of lymphocytes, the vasculature has to grow in a similar way. Increasing blood endothelial cell numbers were already shown (5, 67) and are supplied by special precursors-like endothelial cells. Brulois and colleagues (65) found a capillary endothelial cell subset which remarkably displays features associated with multipotent progenitor cells and participates in vascular neogenesis. This overall reorganization of LNs during inflammation is well described (68–71) and might explain the need for a larger area for the vasculature to enter or exit the organ for an effective remodeling of vascular structures. Multiple small arterioles spread over a larger area to cover more space compared to one large artery formerly described for the hilus morphology. With greater space covered by small arterioles it might be easier during inflammation to develop new capillaries needed for the growing volume of the LN. Furthermore, the less dense capsule of the upper surface which defines the hilus region may allow easier lymph node expansion and vascular remodeling during inflammation. Additionally, it is proposed that LN lobes might function as independent antigen surveillance centers (1, 3). This involves separate swelling of lobes with individual contact to drained antigens and might require a more evenly distributed vasculature to only remodel certain parts rather than the whole vascular tree and also allows for a differentiated regulation of blood flow within the organ.

### High endothelial venules are distributed in close proximity to B-cell follicles

In addition to the reorganization of LNs during inflammation, a major function of the vasculature is lymphocyte homing and recruitment (72). That takes place at specialized HEVs. Von Andrian (10) elucidated the organization of venules including those HEVs by categorizing them into different orders. HEVs are designated to order three to five venules showing a typical jump in lumen diameter over the transition from capillary to proper HEV. Based on his classification, we were able to identify HEVs in reconstructions of our SRµCT images and were able to prove identifications by correlation with SEM data (see **Figure 3**). In accordance with his findings, we identified order three HEVs as situated within the paracortical region of the LN while order four to five HEVs were found in close proximity to follicles within the interfollicular space and at the T-cell-B-cell border throughout the follicular margin. Indispensable for lymphocyte trafficking, HEVs are crucial for maintaining immunological function of LNs. Directional guidance out of the blood stream into LN compartments is provided by secretion of different chemoattractants. In addition, our high-resolution images reveal the spatial arrangement of HEVs in a much closer proximity to B-cell follicles than previously reported. Surprisingly, we not only identified HEV close to, but also detected a dense arrangement of HEVs around the follicles. During steady state of the LN, follicular dendritic cells (FDC) within the follicle produce the chemoattractant CXCL13, which is crucial for coordinating B-cell trafficking from the HEVs towards B-cell follicles. The conduit network forms a dense structure around HEVs and connects HEVs with follicle borders (73) Inside the follicle, the conduit is relatively sparse, whereas the border between the B-cell and T-cell area consists of a dense conduit (73, 74). It was shown that CXCL13 exists as an immobilized form, binding to extracellular matrix components of the conduit but also can diffuse as a soluble when cleaved (75, 76). It was shown that B-cells migrate along the conduit, thus potentially using bounded CXCL13 for guidance (77). Chemoattraction by CXCL13 could be limited due to its slow diffusion rate of 6.2 µm^2^ per second (76) and therefore might be restricted within a close proximity of the follicle. Interestingly, it is thought that HEVs are generally located within the paracortical area of the LN (78) which in return would require B-lymphocytes to migrate long distances through the T-cell zone to reach the B-cell follicles as their destination. Additionally, during inflammation follicles grow in size and displace the surrounding T-cell area. During this displacement, new areas are getting directly associated with the expanding follicle, thus becoming perifollicular regions. This provokes the need for further CXCL13 expression to provide sufficient B-cell migration. A FRC subset, the versatile stromal cells (VSC), can switch on CXCL13 expression during inflammation to sustain B-cell homing into the newly grown areas (79). Based on the spatial arrangement of HEVs in close proximity to the follicles, we hypothesize that not only CXCL13 gradients play an essential role in B-cell homing. It was shown that human as well as murine B-lymphocytes preferably migrate through follicle associated HEVs instead of typical T-cell zone located HEVs (33, 64). These new insights in accordance with our striking 3D analysis of the arrangement of HEVs around B-cell follicles can highlight the migratory behavior of B-cells with a more nuanced view.

### Capillary density in B-cell follicles exceeds that in the deep cortical units of the T-cell zone for exaggerated recruitment of lymphocytes

Capillary distribution within every organ is crucial for sufficient energy and oxygen delivery as well as waste disposal. This is no exception to the murine inguinal LN. We detected a dense capillary network within all LN compartments. To further investigate differences in density, we measured the percentage of capillaries within the area of set volumes. Our data suggest that the capillary density in B-cell follicles and interfollicular regions is about twice as much as in the DCU of the T-cell zone. This contradicts previous reports done with either corrosion cast models or with perfusion of fluorescent markers into LNs (14, 39, 62). However, Kelch and colleagues (39) also showed a higher vascular density for the perifollicular area. For both methods it is likely that the solutions used for perfusion do not reach to the very finest capillaries. This might have resulted in an incomplete picture of the micro vascularization of LNs (38). With high resolution SRµCT scans we were able to identify even the smallest capillaries with an inner diameter down to 5 µm, which made it possible to reveal a section of the entire vascular tree of the LN. Despite our convincing results, it is still unclear why the capillary density within B-cell follicles is much higher compared to the DCU. One explanation for this could be the need for nutrients, energy and oxygen during somatic hypermutation and proliferation of B-cells during an inflammatory state, which exceeds the capabilities of a less dense capillary bed (5). In addition, the oxygen supply seems to play an important role for proper B-cell function and arrangement, as recent studies claim serve B-cell abnormalities during hypoxia in COVID-19 (80). Evidence for another scenario could be found in the migration and homing of lymphocytes through HEVs. In order to transmigrate through the endothelial wall of HEVs, lymphocytes need to get in contact with the endothelium. Within normal blood flow, it is unlikely that lymphocytes can make sufficient contact with the vessel wall to induce rolling adhesion and transmigration. Capillaries can support this procedure. Passing through the lumen of capillaries, lymphocytes make great contact to the capillary wall and slow down. During this process the capillary is blocked for faster flowing cells like erythrocytes. As soon as the lymphocyte reaches a point of lumen extension - like at the transition from capillary to HEV - it gets overtaken by the accumulated erythrocytes behind it. The shear mass of red blood cells pushes the lymphocyte towards the HEV wall and ensures a sufficient contact for adhesion (81, 82). Interestingly, five new subsets of capillary endothelial cells were classified based on single cell RNA sequencing, one of which forms a transitional cell type between capillary endothelial cells and high endothelial cells. These transitional endothelial cells (TrEC) express glycotopes which tether selectively with lymphocytes. This might pre-sort potential lymphocytic candidates for transmigration through following HEVs (65). Additionally, under inflammatory conditions, capillary endothelial cells in association with the blood brain barrier can alter their expression of adhesion molecules in order to allow lymphocyte migration (83). This might as well be true for LNs under high inflammatory inputs. Both of these functional properties for capillaries (forcing a connection between lymphocytes and endothelial cells as well as the selective pre-sorting of lymphocytes who are suitable for transmigration) benefit from a higher capillary density within B-cell follicles. With higher capillary density, more lymphocytes are potentially migrating through the perifollicular HEVs and supporting adequate antigen surveillance and T-/B-cell interactions directly at the place where they are needed.

## Conclusion

Up to now, spatial analysis of different structures within the complex compartmentation of LNs is still contradicting and often lacks a comprehensive view onto the whole organ in a close to native state. We investigated the LN morphology with X-ray phase-contrast imaging with a 2D Talbot array and were able to visualize distinct structural compartments and the vascular network within the LN in an organ-wide scale. Remarkably, a spatial resolution of 1.84 µm, which is comparable or better than what is possible with other methods, was achieved and the procedure required no preparation steps beyond standard fixation routine. We were able to gain insight into the exact entrance and exit routes for blood vessels, which form several branches that spread over approximately one third of the LN surface before entering. Furthermore, we confirmed the spatial correlation of high endothelial venules, the primary site of lymphocyte migration, with B-cell follicles, the primary site of B-cell activation and proliferation. At last, we revealed differences in the degree of capillarization of the deep cortical unit of the T-cell area and the B-cell follicles, being significantly higher for the latter one. Next steps will include the establishment of a complete cryo-workflow that will be maintained during scans to minimize tissue shrinkage and mirror a close to *in vivo* condition. In addition, the application of the method will be optimized for semi-automatic reconstruction to gain the full high throughput potential. As a necessary consequence of this work further studies may focus on the comparison of the steady state with an inflammatory situation to reveal possible alterations in blood vascular networks.

## Data availability statement

The original contributions presented in the study are included in the article/**Supplementary Material**. Further inquiries can be directed to the corresponding author.

## Ethics statement

The mice were bred in the institute’s animal facility at the University of Hamburg. This study was carried out in accordance with the recommendations of the European Union’s and local animal welfare guidelines. Mice were euthanized using isoflurane (5% mixed with 1 L/min O2) before using for experiments. The permission to despatch mice for the purpose of organ extraction was obtained by the Hamburg Authority of Health and Consumer Protection (GZ G21305/591-00.33; Behörde für Gesundheit und Verbraucherschutz, Hamburg, Germany). According to the local laws, an additional ethical approval for animal experiments in the current study was not required.

## Author contributions

Conceptualization: SH, CW. Methodology: SH, JUH, MR, MB, JH. Software: MR, PS. Validation: SH, FS, PS. Formal Analysis: PS, FS, SH. Investigation: SH, PS, FS, JUH, MR, BW, SB, S-CF. Resources: CW, CL, MB, JH. Data Curation: MR, JUH, PS. Writing – Original Draft Preparation: FS, SH, PS. Writing – Review & Editing: SH, CW, JB, MB, JUH, CL, JH, SB, MR, BW, FS, PS. Visualization: PS, SH, FS. Supervision: SH. Project Administration: SH, CW. Funding Acquisition: CW, SH, MB, JUH and JH. All authors contributed to the article and approved the submitted version.

## Funding

This work was supported in part by Deutsche Forschungsgemeinschaft (DFG), Research Training Group, under grant GRK 2274. We also acknowledge provision of beamtime related to the proposal I-20190799 at beamline P05, PETRA III at DESY, a member of the Helmholtz Association (HGF).

## Acknowledgments

We want to thank all colleagues of the animal facility of the University of Hamburg, involved in the housing of the animals needed for this study, for their support. The valuable comments on our manuscript and the help of Frank Friedrich (University of Hamburg) and his team during the tissue processing for resin embedding is highly appreciated. In addition, we are grateful to Mike Hasenberg from the Electron Microscopy Unit at IMCES, University Duisburg-Essen for the opportunity to conduct high resolution SEM experiments. The helpful comments of Hauke S. Günther regarding the initial DESY beamtime proposal are also greatly acknowledged. We would also like to thank Pierre Thibault for providing the UMPA package. Finally, we acknowledge the support during the beam time by Hereon team members Fabian Wilde, Julian Moosmann and Felix Beckmann. This research was supported in part through the Maxwell computational resources, operated at Deutsches Elektronen-Synchrotron DESY, Hamburg, Germany.

## Conflict of interest

The authors declare that the research was conducted in the absence of any commercial or financial relationships that could be construed as a potential conflict of interest.

## Publisher’s note

All claims expressed in this article are solely those of the authors and do not necessarily represent those of their affiliated organizations, or those of the publisher, the editors and the reviewers. Any product that may be evaluated in this article, or claim that may be made by its manufacturer, is not guaranteed or endorsed by the publisher.
