# Supplementary material for: Three-dimensional analyses of vascular network morphology in a murine lymph node by X-ray phase-contrast tomography with a 2D Talbot array
Source: Front Immunol. 2022 Nov 29;13:947961. doi: 10.3389/fimmu.2022.947961 (PMC9745095; doi:10.3389/fimmu.2022.947961)
Supplement: Supplementary file 1 [file DataSheet_1.pdf]

Supplementary video data

Supplement Video S1. Supplementary video corresponding to Fig 2.

Supplement Video S2. Supplementary video corresponding to Fig 4.

Supplement Video S3. Supplementary video corresponding to Fig 5.

Supplement Video S4. Supplementary video corresponding to Fig 6.

Supplement Video S5. Supplementary video corresponding to Fig 7.

Supplement Video S6. Supplementary video corresponding to Fig 9.

Supplement Video S7. Animation of the whole murine lymph node model acquired by means of X-ray phase-contrast tomography with a 2D Talbot array and subsequent manual structure reconstruction. The animation features the whole volume rendering (grey) before and after adjustment of the data histogram. Different camera paths elucidate the outer and inner organization of the lymph nodes vessel architecture in the reconstructed as well as non-reconstructed regions of the lymph node. Efferent (dark green) as well as afferent lymphatic (light green) vessels are present only outside of the lymph node. Blood vessels (arterial system=red; venous system=blue) enter and exit the node at multiple points over the large upper surface area and arch through the medulla and deep cortical unit (yellow) up to the follicles (mint green) which are distributed on the bottom side of the node. Capillaries are present in any compartment of the lymph node, but seem to be arranged in a denser constellation within the follicles compared to the deep cortical unit.
