# Supplementary material for: Three-dimensional analyses of vascular network morphology in a murine lymph node by X-ray phase-contrast tomography with a 2D Talbot array
Source: Front Immunol. 2022 Nov 29;13:947961. doi: 10.3389/fimmu.2022.947961 (PMC9745095; doi:10.3389/fimmu.2022.947961)
Supplement: Supplementary file 2 [file Table_1.pdf]

Supplement Table S1. Spatial comparison between branching order III to V venules of the deep cortical unit with venules situated at the medullary margins as well as in the interfollicular regions.

	Order III		Order IV		Order V	
	Segment Count	Volume (mm^3)	Segment Count	Volume (mm^3)	Segment Count	Volume (mm^3)
DCU	28	0.0013	52	0.0014	33	0.0006
MM and IFR	59	0.004	101	0.003	69	0.0013
Ratio	2.11	2.98	1.94	2.14	2.09	2.04

The segment count and the volume of venules were used as comparative parameters between the deep cortical unit (DCU) and the venules located at the medullary margins (MM) and the interfollicular regions (IFR). Segment counts were obtained via skeletonization and volumes (mm^3) were calculated from their respective voxel counts. The ratio between the two groups was calculated by dividing the value of the MM and IFR group by the DCU group. Overall, the comparative parameters were approximately twice as high in the MM and IFR group than in the DCU group.
