# Supplementary material for: Three-dimensional analyses of vascular network morphology in a murine lymph node by X-ray phase-contrast tomography with a 2D Talbot array
Source: Front Immunol. 2022 Nov 29;13:947961. doi: 10.3389/fimmu.2022.947961 (PMC9745095; doi:10.3389/fimmu.2022.947961)
Supplement: Supplementary file 3 [file Image_1.pdf]

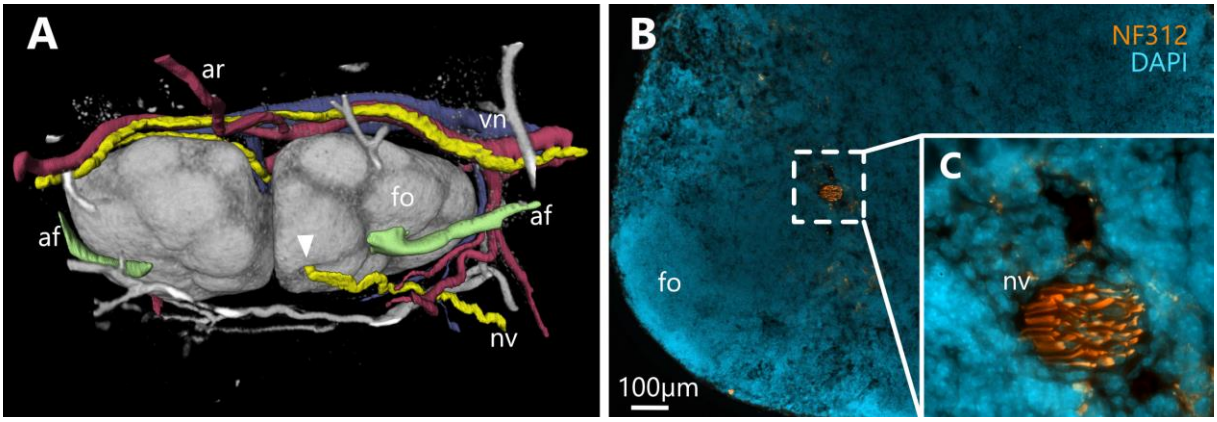

Supplement Fig S1. A big nerve bundle is penetrating the lymph node, observed in two different murine inguinal lymph nodes. (A): Volume rendering of an image stack acquired by means of X-ray phase-contrast tomography with a 2D Talbot array. Depicted is an antero-basal view on a SRµCT tomography of a lymph node (LN), which is subdivided into two lobes, and manually reconstructed blood as well as lymphatic vessels and big nerve bundles. One nerve bundle (yellow) approaches the LN at the entrance point of blood vessels and projects superficially across the LN towards the intersection of the two lobes where it penetrates the whole organ and leaves on the opposite side (arrowhead). (B): Immunofluorescence image displays immunoreactivity against anti-pan neurofilament (NF312) showcasing a big nerve of approximately 50 µm in diameter passing through the LN. Counterstaining with DAPI as a nuclear marker suggests a similar spatial orientation of the nerve bundle as displayed in panel A. The cell density where the nerve bundle could be located has the appearance of a loose cell compartment corresponding to the medullary regions of the organ. Still, the nerve bundle projects through the organ in proximity to the follicles. (C): Higher magnification of the insert in panel B revealing single axon fibers within the nerve. Abbreviations: Afferent lymphatic vessel=af, arterial system=ar, B-cell follicle=fo, nerve=nv, venous system=vn.
