# Supplementary material for: Three-dimensional analyses of vascular network morphology in a murine lymph node by X-ray phase-contrast tomography with a 2D Talbot array
Source: Front Immunol. 2022 Nov 29;13:947961. doi: 10.3389/fimmu.2022.947961 (PMC9745095; doi:10.3389/fimmu.2022.947961)
Supplement: Supplementary file 4 [file Image_2.pdf]

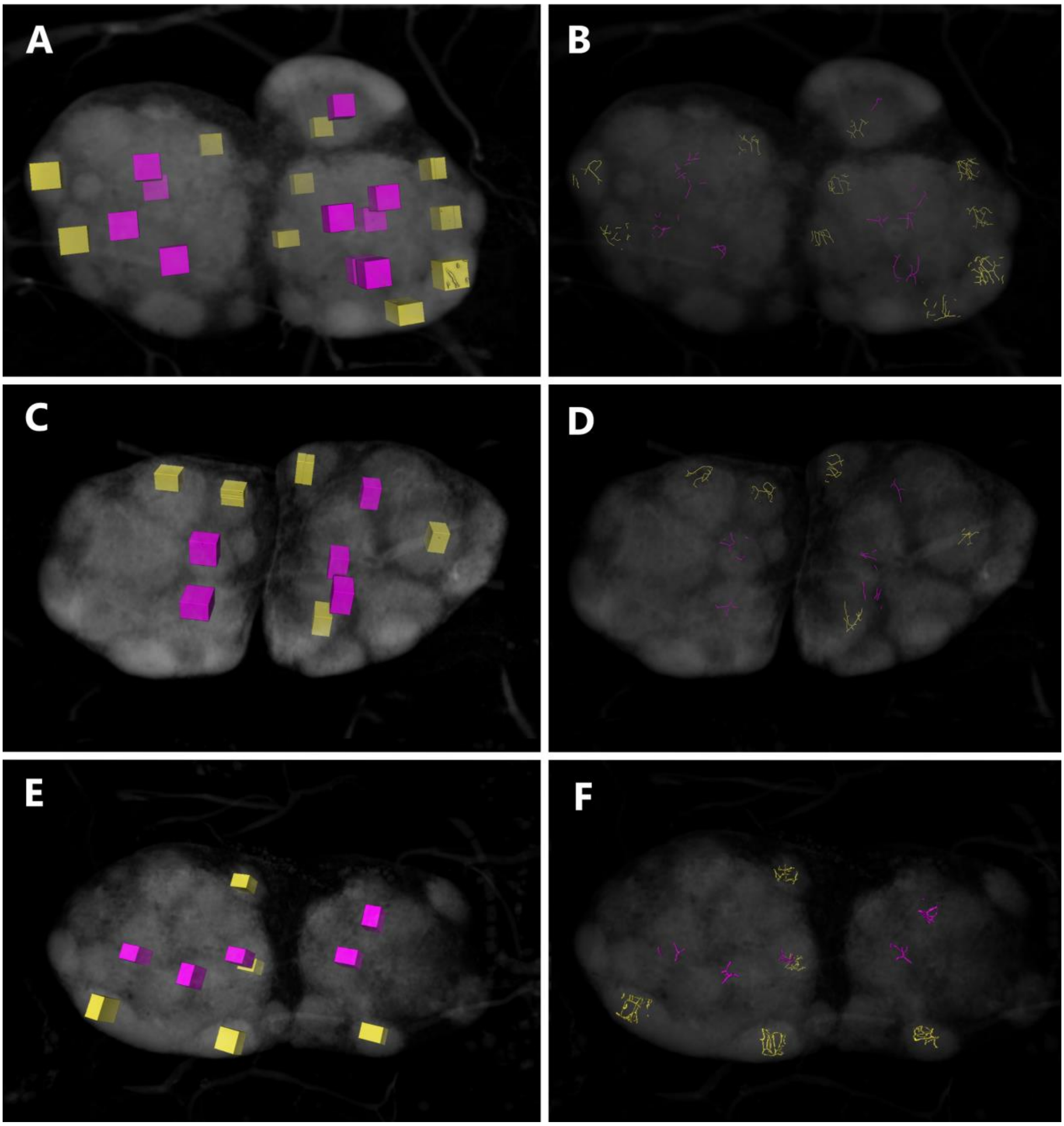

Supplement Fig S2. Quantification of capillary densities in different compartments of a murine inguinal lymph node. (A-F): Volume rendering of an image stack acquired by means of X-ray phase-contrast tomography with a 2D Talbot array. (A,C,E): The lymph nodes (LN) are displayed from the top view. Three different inguinal LNs are displayed in low opacity. Volume of interest (VOI) boxes are randomly placed inside the corresponding compartments being magenta boxes for the deep cortical unit and yellow boxes for the B-cell follicles for capillary density quantification. (B,D,F): The VOI boxes from A, C and E are masked out, revealing the reconstructed and subsequently skeletonized capillary network.
