# Supplementary material for: Three-dimensional analyses of vascular network morphology in a murine lymph node by X-ray phase-contrast tomography with a 2D Talbot array
Source: Front Immunol. 2022 Nov 29;13:947961. doi: 10.3389/fimmu.2022.947961 (PMC9745095; doi:10.3389/fimmu.2022.947961)
Supplement: Supplementary file 5 [file Image_3.pdf]

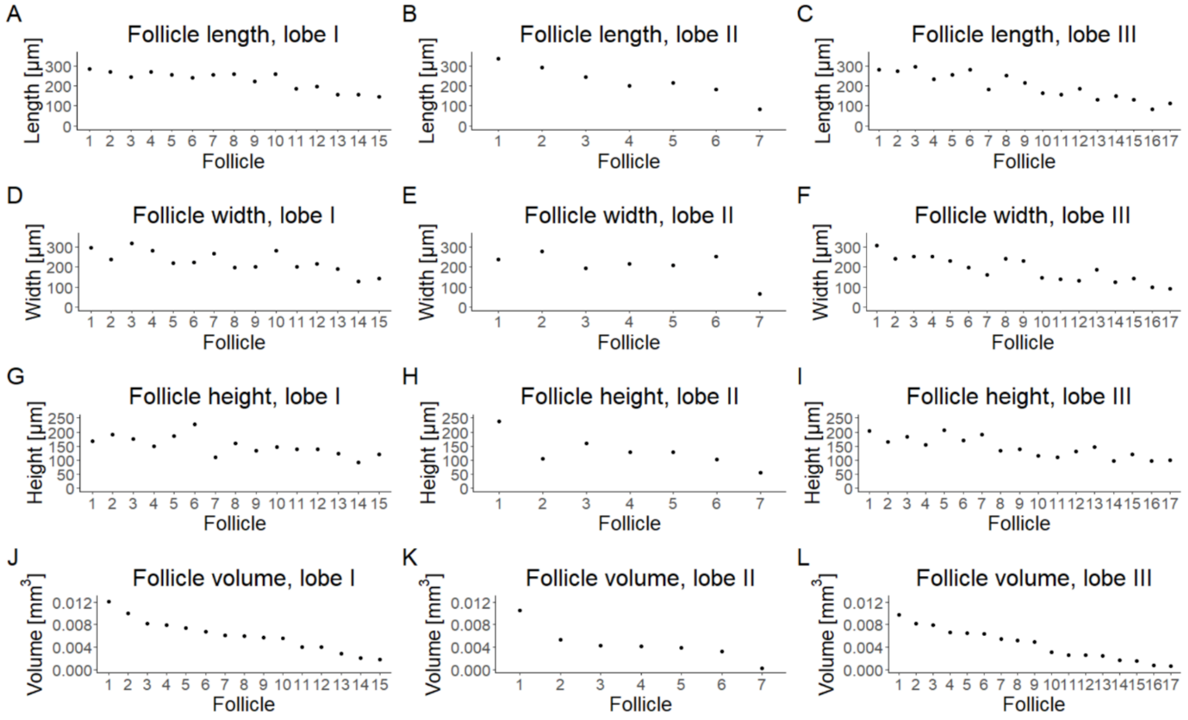

Supplement Fig S3. Different measurements of the B-cell follicle size in different lobes of a murine inguinal lymph node. (A-I): Follicles of the three different lobes of one lymph node (LN) (see Fig 5) were measured in length, width and height using ImageJ. Arranged in columns are the measurements for the respective lobe. Each point reflects the measurement value of one follicle. Follicles are ordered by declining volume. Length dimension describes the dimension running parallel to the Hilus region, while width describes the dimension running vertical to the hilus. Height describes the dimension running centripetally to the LN. Between the lobes, follicle measurements show similar range and tendency. Height displays among the measurements the least variation and lowest values. (J-L): Volumetric measurements of the follicles calculated via the voxel counts in Amira. Follicle volumes between the lobes are in a similar size range.
